# Spatial–Temporal Trends of Cancer Among Women in Central Serbia, 1999–2021: Implications for Disaster and Public Health Preparedness

**DOI:** 10.3390/healthcare13172169

**Published:** 2025-08-30

**Authors:** Emina Kričković, Vladimir M. Cvetković, Zoran Kričković, Tin Lukić

**Affiliations:** 1 Faculty of Geography, University of Belgrade, Studentski trg 3/III, 11000 Belgrade, Serbia; emina.krickovic@gef.bg.ac.rs; 2 Department of Disaster Management and Environmental Security, Faculty of Security Studies, University of Belgrade, Gospodara Vučića 50, 11040 Belgrade, Serbia; 3 Scientific-Professional Society for Disaster Risk Management, Dimitrija Tucovića 121, 11040 Belgrade, Serbia; 4 Safety and Disaster Studies, Department of Environmental and Energy Process Engineering, Technical University of Leoben, Franz Josef-Straße 18, 8700 Leoben, Austria; 5 Military Geographical Institute—“General Stevan Bošković”, Ministry of Defence of the Republic of Serbia, Mije Kovačevića 5, 11000 Belgrade, Serbia; zoran.krickovic@vs.rs; 6 Department of Geography, Tourism and Hotel Management, Faculty of Sciences, University of Novi Sad, Trg Dositeja Obradovića 3, 21000 Novi Sad, Serbia; tin.lukic@dgt.uns.ac.rs; 7 Ruđer Bošković School, Kneza Višeslava 17, 11000 Belgrade, Serbia

**Keywords:** emergency preparedness, breast, cervical, uterine, colorectal, bladder, ovarian, pancreatic, lung and bronchus cancers, Mann–Kendall Test

## Abstract

Background/Objectives: Cancer is a major public health burden in Serbia and a factor influencing long-term disaster readiness by straining health system capacity. This study examined spatial and temporal trends in incidence and mortality for eight major cancers among women in Central Serbia (1999–2021) to inform targeted prevention and preparedness strategies. Methods: Standardised rates from national datasets were analysed using the Mann–Kendall trend test and Sen’s slope estimator. Geographic disparities were mapped in ArcGIS Pro 3.2. Mortality trends were assessed only for statistically reliable series. Results: Breast cancer incidence increased in six counties, while cervical cancer declined in several areas, likely reflecting screening success. Colorectal, bladder, pancreatic, and lung and bronchus cancers showed rising incidence; lung and bronchus cancer mortality increased in 16 counties, indicating growing demand for chronic respiratory care. These shifts may reduce surge capacity during disasters by increasing the baseline burden on healthcare infrastructure. Regional disparities highlight uneven system resilience. Conclusions: Aligning cancer control measures—especially for high-burden cancers like lung—with emergency preparedness frameworks is essential to strengthen health system resilience, particularly in resource-limited regions.

## 1. Introduction

Health risk trends, particularly those associated with chronic and non-communicable diseases, are becoming a critical aspect of public health planning and disaster preparedness [1,2,3,4,5]. Although sudden hazards, such as floods, earthquakes, or pandemics, often receive priority in disaster management systems, gradual shifts in health, including increasing cancer rates and other chronic conditions, can subtly yet significantly influence the vulnerability of populations [6,7,8,9]. If unaddressed, these risks can worsen the effects of crises, reduce emergency response effectiveness, and disproportionately affect vulnerable groups, such as women [10,11,12]. For example, different natural hazards have the potential to increase the risks of infectious diseases, malnutrition, cardiovascular issues, and respiratory illnesses [10,13]. This becomes particularly evident in situations where public health conditions are already deteriorating, access to healthcare is limited, or living conditions are worsening [11,12,14]. Therefore, overburdened or damaged public health infrastructure can greatly slow down emergency response and reduce the level of care quality [10]. Moreover, it is clear that higher rates of chronic diseases (e.g., cancer, diabetes, depression) are directly associated with increased vulnerability, particularly among older adults [15,16].

Concerning health risk trends, cancer remains one of the foremost health threats to women globally. This trend exhibits significant variability in incidence and mortality across different cancer types, age groups, geographic regions, and socioeconomic statuses [1,2,6,8]. Globally, the burden of female cancers remains substantial. In 2021, the total number of new cases was estimated at approximately 1,013,475, corresponding to an age-standardised incidence rate of 50.7 per 100,000 women [17].

Breast cancer continues to be the most commonly diagnosed cancer in women, with increasing incidence and mortality rates noted in many areas, especially in low- and middle-income nations and among women of childbearing age [17,18,19,20]. Forecasts suggest that by 2040, new cases in this age group could rise by almost 48% [18]. Conversely, cervical cancer remains the second most prevalent cancer among women in numerous developing countries, showing a strong inverse relationship between its incidence and socioeconomic status [20,21]. Nonetheless, enhanced availability of HPV vaccination and screening has led to decreased rates in specific areas [19,21]. Additionally, while ovarian and endometrial (uterine) cancers are less common than breast or cervical cancer, they have been trending upward in various populations.

Similarly, according to data from the World Cancer Research Fund, in 2022, breast cancer, along with tracheal, bronchial, and lung cancers, remained the most prevalent malignancies among the global female population [22]. Additionally, colorectal, cervical, ovarian, and uterine cancers are also highly prevalent [22].

Extensive population-based research consistently reveals that cancers specific to females—namely breast, cervical, ovarian, and uterine cancers—pose a serious and growing global health issue. Over the last thirty years, both the incidence and mortality rates linked to these cancers have generally risen, with breast cancer being the most common and responsible for the most significant number of new cases and fatalities among women globally [20,23,24]. Breast, ovarian, and uterine cancer incidence rates are positively associated with higher socioeconomic and human development indices (SDI/HDI). In contrast, cervical cancer incidence is significantly greater in areas with lower socioeconomic status [20,24,25].

Many modifiable risk factors significantly impact the global cancer burden in women. Among these are obesity, tobacco use, alcohol consumption, lack of physical activity, and HPV infection [26,27,28]. Dietary patterns significantly impact health: diets rich in pro-inflammatory and insulinogenic foods correlate with increased cancer risk, whereas following higher-quality diets is associated with a lower risk of breast, endometrial, and colorectal cancers, especially in postmenopausal women [28]. Significant socioeconomic and geographic disparities persist. Cancer mortality is particularly elevated in low- and middle-income nations, primarily because of restricted access to early detection, screening, and treatment solutions [20,29].

There are notable geographic and socioeconomic differences in cancer distribution. Higher rates of breast and ovarian cancers are found in urban and developed areas, whereas cervical cancer is more common in rural and economically disadvantaged region [20,25,30]. Age-standardised incidence rates for breast, ovarian, and uterine cancers generally rise with socioeconomic development, whereas cervical cancer shows a contrary trend [20,24,25].

The rising cancer burden and notable regional disparities highlight the pressing demand for focused public health interventions. Successful strategies include improved cancer screening programmes, widespread HPV vaccination campaigns to avert cervical cancer, and initiatives encouraging lifestyle changes. Efficient resource distribution should consider local epidemiological factors and socioeconomic contexts to address these global health challenges effectively [20,24,25].

This burden is particularly pronounced in low- and middle-income countries, where cervical cancer remains a leading cause of cancer-related mortality among women. Shrestha et al. [31] examined the prevalence, incidence, and mortality rates of cervical cancer in these settings, highlighting persistent disparities in outcomes. Further emphasising these disparities, Olson et al. [32] evaluated cervical cancer screening programmes and guidelines, identifying key barriers to effective implementation—such as financial constraints, limited public awareness, geographic inaccessibility, and sociocultural beliefs—which continue to hinder early detection and timely treatment in vulnerable populations.

Yi et al. [20] conducted a comprehensive population-based study that analysed epidemiological trends in female-specific cancers—namely breast, cervical, ovarian, and uterine cancers—across global, regional, and national levels from 1990 to 2019. Similarly, Sun et al. [17] focused on the patterns and trends of cancer among women of childbearing age, covering 204 countries and territories from 1990 to 2021. These studies highlight the dynamic nature of cancer epidemiology among women and emphasise the importance of monitoring temporal trends to inform public health strategies. In a related methodological context, Chen et al. [33] demonstrated the applicability of the Mann–Kendall–Sneyers test for identifying change points in time series data, using COVID-19 trends in the United States as a case study.

Research at the intersection of cancer epidemiology and disaster or public health preparedness has gained increasing scholarly attention over the past decade [34,35,36,37]. This multidisciplinary domain brings together public health professionals, oncologists, gerontologists, and disaster risk management specialists, often working within academic, clinical, and governmental institutions [34,35,36,37,38,39,40,41,42,43].

Several leading researchers have made significant contributions to this emerging field. For instance, Prohaska and Peters [37] explored how natural hazards disproportionately affect older adults, pointing out their heightened cancer risk and susceptibility to disruptions in care. Nogueira, Sahar, and their team [42] examined the role of U.S. National Cancer Institute (NCI)—designated Cancer Centres in promoting emergency preparedness for oncology patients, particularly in the context of climate-related threats. Likewise, Jim et al. [35] investigated how extreme weather events, including hurricanes, impact cancer survivorship, emphasising the wider social and structural factors influencing health outcomes.

In ageing societies, Kinoshita, Gonda, and colleagues [36] showcased case studies from Japan’s Noto Peninsula, revealing how cancer care is handled in super-aged populations during disasters. Their research highlights the necessity for adaptable care models that consider both demographic changes and environmental threats [36].

According to the World Cancer Research Fund, Serbia is among the countries with high cancer rates, ranking 60th globally in newly diagnosed cancer cases among women and 57th in cancer-related mortality in 2022 [22]. In the territory of central Serbia, studies on cancers have been conducted [44,45,46,47,48,49,50,51,52,53,54]. Sipetic-Grujicic et al. [55] analysed trends in breast cancer incidence and mortality among males and females in Central Serbia from 1999 to 2009. Also, Mihajlović et al. [56] examined cancer incidence and mortality rates in Serbia from 1999 to 2009, and Perišić et al. [57] initiated cervical cancer screening programmes in Serbia. Similarly, Naumović et al. [58] examined cervical cancer mortality in Serbia (1991–2011), while Stojanović et al. [52] analysed incidence trends of primary gynaecological cancers in Central Serbia (2003–2018).

This study aims to analyse trends in eight types of cancer—breast, cervical, uterine, colorectal, bladder, ovarian, pancreatic, and lung and bronchial—in the female population (1999 to 2021) across eighteen counties in Central Serbia. The study does not investigate the causes of cancer in women but instead focuses on analysing temporal trends. Examining cancer trends over time is of great importance as it identifies increasing or decreasing patterns, informs public health planning, and contributes to the evaluation of existing prevention and screening programmes. The literature highlights various ongoing challenges in cancer care following disasters, including interruptions in treatment protocols, a lack of qualified healthcare personnel, and constraints in health system infrastructure [36,39,40]. These disruptions particularly impact high-risk groups, like cancer survivors and elderly patients, who may face increased morbidity and mortality due to delays or interruptions in their care [35,36,37].

## 2. Materials and Methods

The analysis was based on standardised incidence and mortality rates for malignant cancers in females. Mortality data for uterine and bladder cancers were excluded due to a lack of statistically reliable information covering the entire study period. Cancer data were obtained from publicly available reports published by the Institute of Public Health of Serbia, “Dr Milan Jovanović Batut,” specifically “Cancer Incidence and Mortality in Central Serbia from 1999–2015” and “Malignant Cancers in the Republic of Serbia 2016–2021.” [59,60,61,62,63,64,65,66,67,68,69,70,71,72,73,74,75,76,77,78,79,80]. Malignant tumours were classified according to the International Classification of Diseases, Tenth Revision (ICD-10, codes C00–C96), and the International Classification of Diseases for Oncology, Third Edition (codes 8000/3–9941/3), as defined by the World Health Organisation [80]. This study is an ecological, geographical-medical, retrospective time-trend analysis based on aggregated cancer incidence and mortality data from Central Serbia for the period 1999–2021.

The Institute of Public Health of Serbia “Dr Milan Jovanović Batut” oversees the management of the Cancer Registry for the Republic of Serbia. The Serbian Cancer Register contains data on the personal characteristics of new cases and deceased individuals, the potential occurrence of multiple primary cancers, the date of diagnosis, diagnostic methods used, and cancer characteristics such as primary and secondary anatomical location, histological type, and stage, disease outcome, and information about the healthcare institution reporting the malignancy [47,59]. Although the register contains the aforementioned data, not all of it is available in the open-source dataset used for this analysis. These data were compiled and organised in Excel spreadsheets and subsequently processed in Python 3.9.11 for Mann–Kendall trend analysis.

It is important to note that the Statistical Office of the Republic of Serbia does not include data for the Autonomous Province of Kosovo and Metohija, as no official data have been available from this region since 1998 [45].

**Figure 1:**
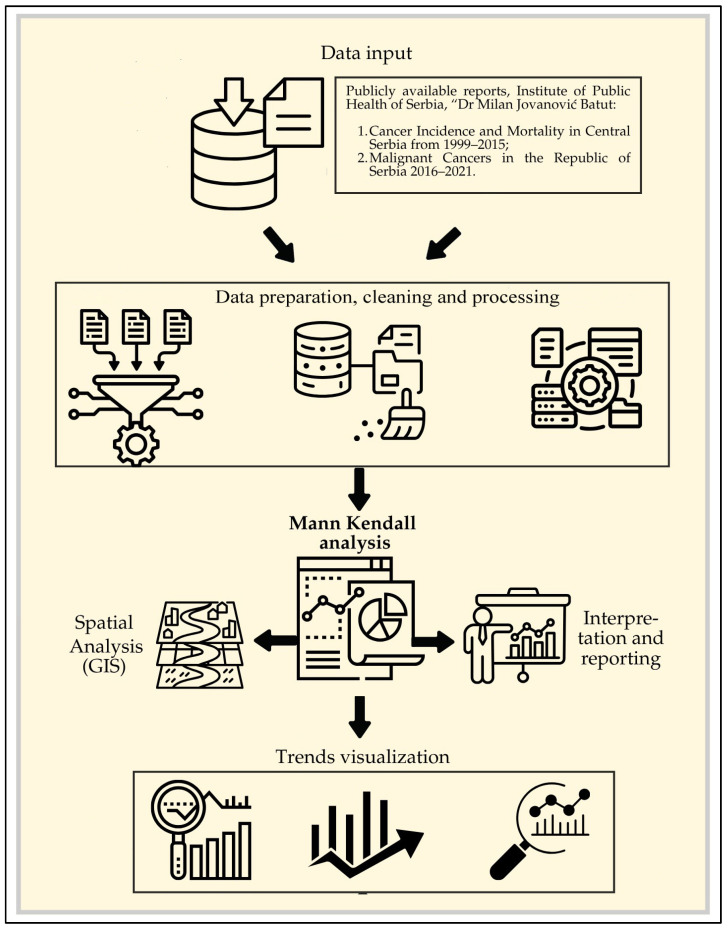
Schematic representation of the research process.

Figure 1 presents the research framework, illustrating the workflow from data collection and processing to the application of software for Mann–Kendall (MK) analysis and the presentation of results.

The MK test [81,82,83,84] is designed to evaluate statistically whether a variable exhibits a consistent upward or downward trend over time. A monotonic trend indicates a steady increase or decrease in the variable across the period, though the trend itself does not necessarily have to follow a linear pattern. The MK trend test is highly versatile, as it can be applied to various types of data [85]. To perform the test effectively, a minimum of ten time series observations is required [85].

According to the MK test, two hypotheses were tested: H_0_ (null hypothesis), where there is no trend in time series, and the alternative hypothesis (H_a_), where there is a statistically significant trend in time series, for the selected significance level (α) [86]. The probability (*p*) was calculated to determine the level of significance of the hypothesis [86]. Sen’s nonparametric estimator of slope utilises a linear model for estimating trend magnitude [81,87]. The statistical significance of the observed trends was defined at the 95% and 99% levels [87].

The MK test is applicable when the time series data ($x_{i}$) adheres to a specific underlying model. This model evaluates trends by analysing the sequence of data points in the series [88,89]

(1) $$S=\sum_{i=2}^{n}\sum_{j=1}^{i-1}sign(x_{i}-x_{j})$$

where ${sign(x}_{i}-x_{j})$ is

(2) $$sign\left(x\right)=\left\{\begin{array}{cc} 1, & for\;x_{i}-x_{j}>0\\ 0, & for\;x_{i}-x_{j}=0\\ -1, & for\;x_{i}-x_{j}<0 \end{array}\right.$$

The statistic *S* tends to normality for large *n*; with mean and variance defined as follows [88,89,90]:

(3) E(S)=0

(4) $$V\left(S\right)=1/18\left[n\left(n-1\right)\left(2n+5\right)-\sum_{P=1}^{q}t_{P}(t_{P}-1)(2t_{P}+5)\right]$$

where *n* is the length of the times-series, *t_P_* is the number of ties for the *p* value and *q* is the number of tied values (i.e., equals values). The second term represents an adjustment for tied or censored data. The standardised test statistic *Z* is given by [88,89,90]:

(5) $$Z=\left\{\begin{array}{c} \frac{S-1}{\sqrt{Var(S)}}\;if\;S>0,\\ 0\;if\;S=0,\\ \frac{S+1}{\sqrt{Var(S)}}\;if\;S<0, \end{array}\right.$$

The *Z* value is used to assess whether a trend in the data is statistically significant. The non-parametric Mann–Kendall (MK) trend test and Sen’s slope estimator are highly effective for analysing temporal trends in geospatial medical geography [47,88,89], especially for female-specific cancers such as breast, cervical, and ovarian cancer. MK detects monotonic trends without assuming data normality, making it robust to non-normal distributions, missing values, irregular time intervals, and outliers—common features of epidemiological datasets. Its rank-based approach captures subtle, long-term increases or decreases in cancer rates, even when short-term fluctuations occur due to changes in reporting or population shifts.

Sen’s slope complements MK by quantifying the magnitude of change in a manner resistant to extreme values, providing clear estimates of the annual or multi-year rate of change. Applied to spatially stratified data, these methods reveal localised variations and long-term dynamics across different geographic units [81,82,83,84,85,86,87,88,89,90]. In cancer statistics, this approach enables the reliable detection of significant temporal changes, the mapping of emerging hotspots, and the identification of regions where incidence is escalating or declining. Such evidence is essential for targeting awareness campaigns, optimising screening programmes, and guiding resource allocation to improve women’s health outcomes.

This analysis was performed using Python within the PyCharm 2023 Community Edition environment. pyMannKendal is a pure Python implementation of non-parametric Mann–Kendall trend analysis, which combines almost all types of Mann–Kendall tests [91]. Currently, this package has 11 MK tests and 2 Sen’s slope estimator functions [91].

For geospatial analysis and presentation of results, ArcGIS Pro 3.2 was used. Data used in the geospatial database were downloaded from open data of the National Spatial Data Infrastructure (NSDI) (https://download.geosrbija.rs/, accessed on 10 November 2024). This portal is under the authority of the Republic Geodetic Authority (RGZ). The downloaded data were in UTM coordinates using the WGS84 ellipsoid.

### Demographic Characteristics

Table 1 contains general population statistics regarding population number and average age by sex. Seventy-three percent of Serbia’s total population lives in Central Serbia [47,92]. According to the 2022 census, the population of Central Serbia was 4,906,773 [47,93] out of which 2,520,534 were women. In Belgrade region 1,681,405 people were recorded (886,992 were women), while the southern part of Serbia had 3,225,368 inhabitants [47,93] (1,633,542 were women). This included 1,819,318 in the Šumadija and Western Serbia region, and 1,406,050 in the Southern and Eastern Serbia region [47,93], out of which were 923,478 and 710,064 women, respectively. Regarding the percentage of women in the population, all counties are below Serbia (51.4%) ranging from 49.5% (Pirotski and Toplički counties) to 52.8% (Beogradski county) [47,93].

The average age of the population in 2022 was 42.7 years in the Belgrade region and 44.5 years in the southern regions (44.3 years in the Šumadija and Western Serbia region, and 44.9 years in the Southern and Eastern Serbia region) [47,93]. Regarding average age in women, it ranges from 41.7 in Raški county, which is below Serbian women’s average age (45.2), to 50.2 in Zaječarski county.

Serbia ranks among the most demographically aged countries in the world [47,94]. The ageing of the total population in Serbia has been an ongoing process for more than 50 years, since the end of the 1960s when the population was demographically the youngest [47,94]. This ageing process has intensified, as indicated by data from the last four censuses (1991, 2002, 2011, and 2022) [47,94]. The average age has been increasing, the number of young people (under 15 years) has declined, and the population aged 85 and older has nearly doubled [47,94].

## 3. Results

### 3.1. Breast Cancer

**Figure 2:**
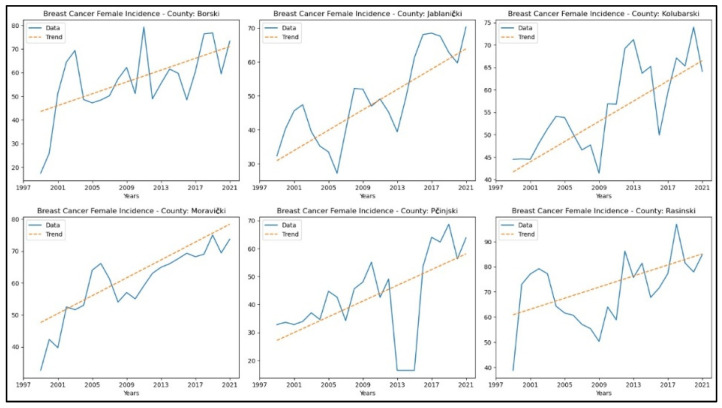
Mann–Kendall analysis of breast cancer incidence rates.

Appendix A reveals increasing trends in breast cancer incidence rates in six counties—Borski, Jablanički, Kolubarski, Moravički, Pčinjski, and Rasinski. In contrast, the remaining counties did not exhibit any notable trends. The graphical representation of this analysis is shown in Figure 2.

**Figure 3:**
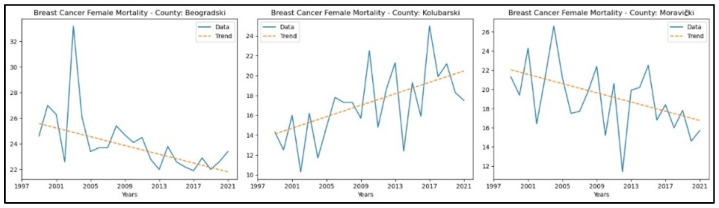
Mann–Kendall analysis of breast cancer mortality rates.

When analysing mortality rate trends for this cancer, an increasing trend was observed in one county, Kolubarski. In contrast, a decreasing trend was observed in Beogradski and Moravički counties. The graphical representation of this analysis is shown in Figure 3.

### 3.2. Cervical Cancer

**Figure 4:**
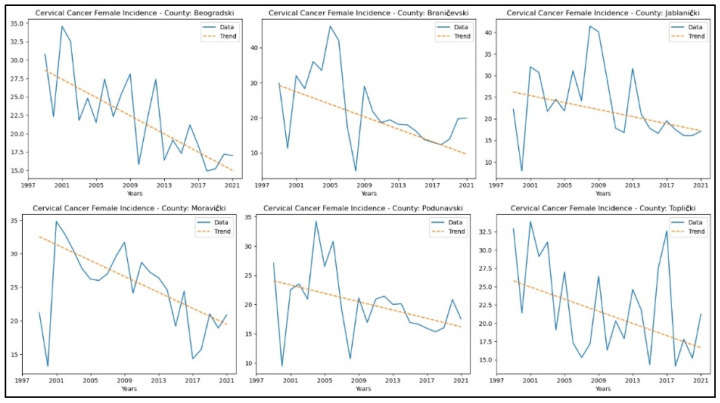
Mann–Kendall analysis of cervical cancer incidence rates.

From Appendix B, it is evident that the counties demonstrating a decreasing trend in cervical cancer incidence rates are Beogradski, Braničevski, Jablanički, Moravički, Podunavski, and Toplički counties. The graphical representation of this analysis is shown in Figure 4.

**Figure 5:**
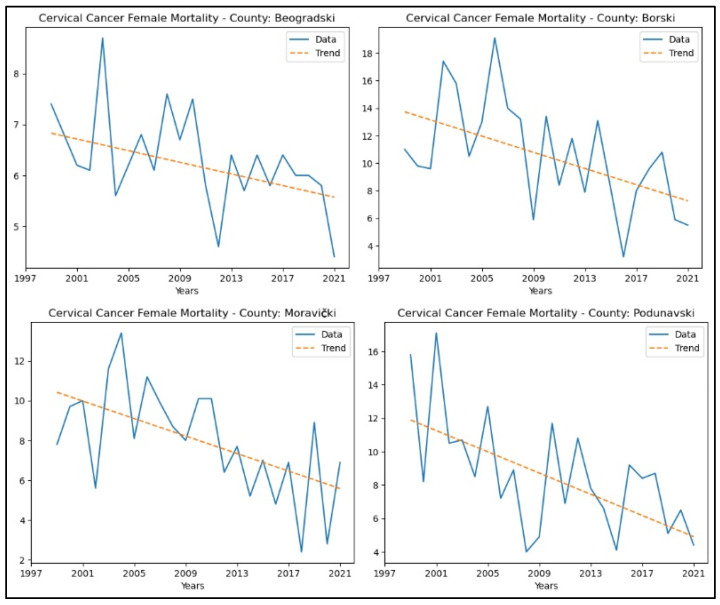
Mann–Kendall analysis of cervical cancer mortality rates.

Regarding mortality rates, the following counties showed a decreasing trend in the number of deaths: Beogradski, Borski, Moravički, and Podunavski. No significant trends were observed in the other counties. The graphical representation of this analysis is shown in Figure 5.

### 3.3. Lung and Bronchus Cancer

**Figure 6:**
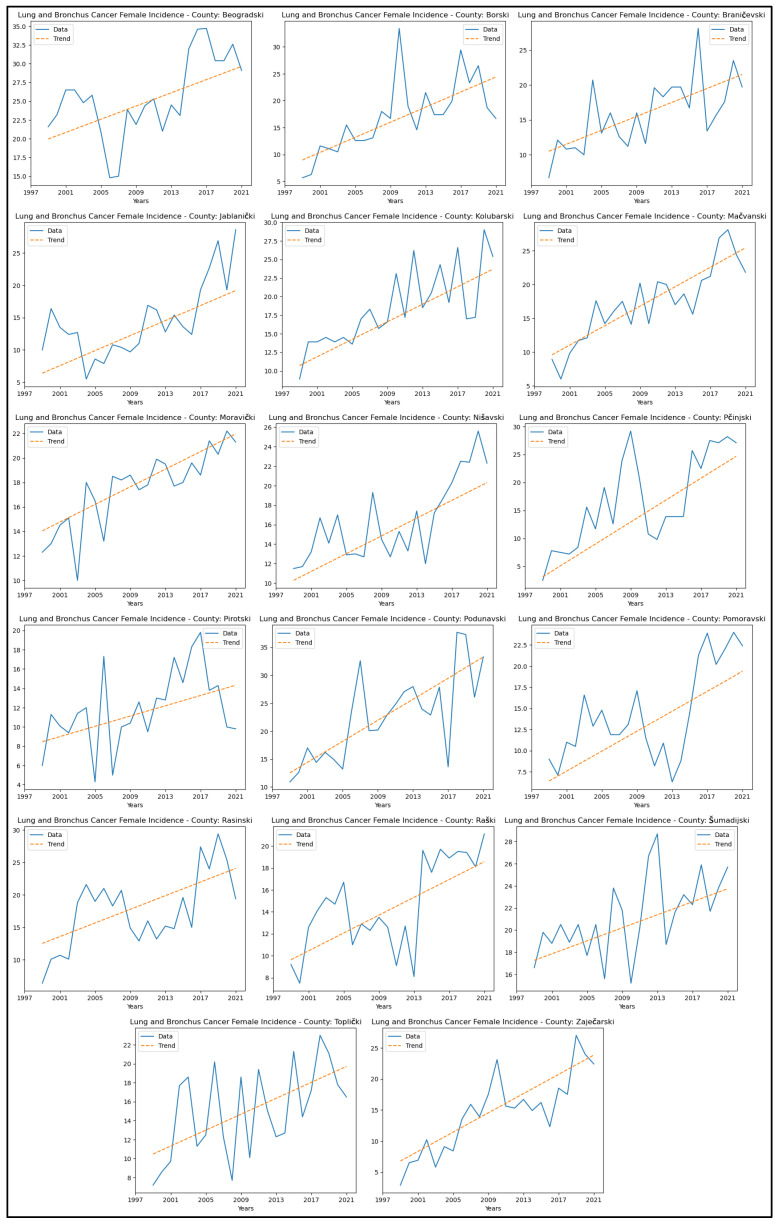
Mann–Kendall analysis of lung and bronchus cancer incidence rates.

Appendix C reveals increasing trends in lung and bronchus cancer incidence rates in seventeen counties—Beogradski, Borski, Braničevski, Jablanički, Kolubarski, Mačvanski, Moravički, Nišavski, Pčinjski, Pirotski, Podunavski, Pomoravski, Rasinski, Raški, Šumadijski, Toplički, and Zaječarski. The graphical representation of this analysis is shown in Figure 6.

**Figure 7:**
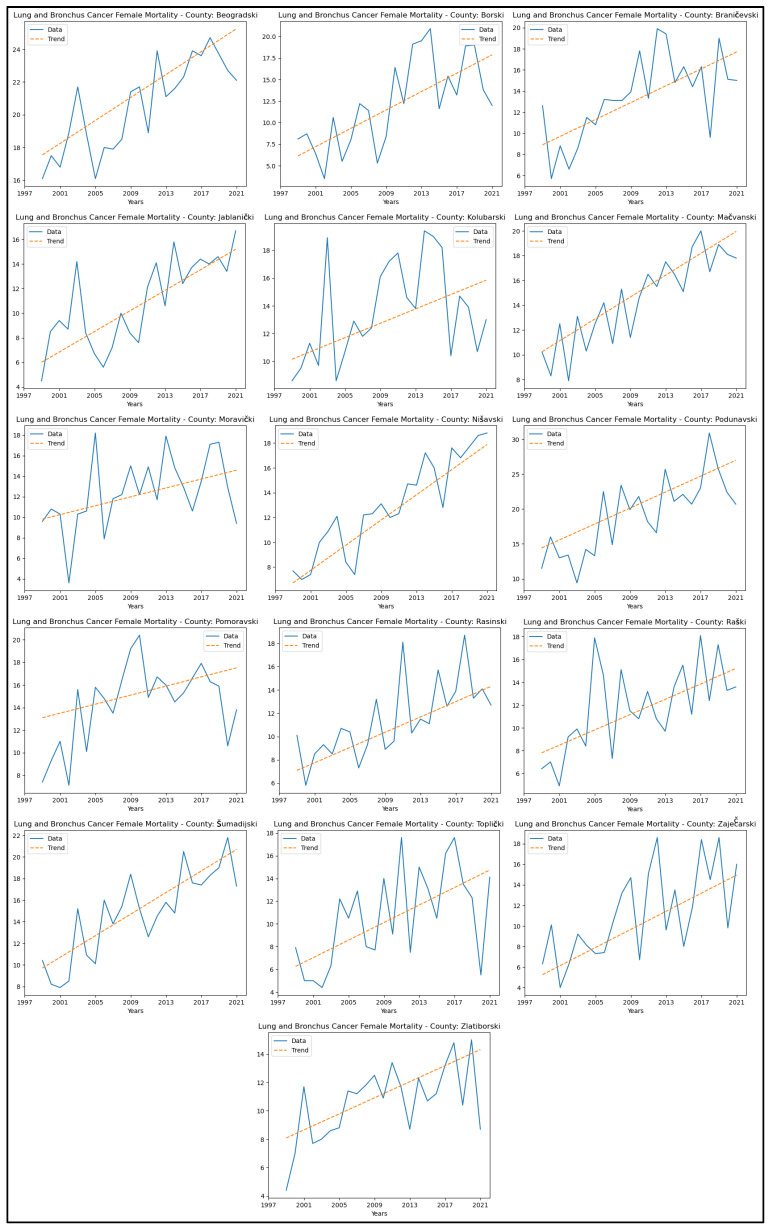
Mann–Kendall analysis of lung and bronchus cancer mortality rates.

When analysing mortality rate trends for lung and bronchus cancer, an increasing trend was observed in sixteen counties: Beogradski, Borski, Braničevski, Jablanički, Kolubarski, Mačvanski, Moravički, Nišavski, Podunavski, Pomoravski, Rasinski, Raški, Šumadijski, Toplički, Zaječarski, and Zlatiborski. The graphical representation of this analysis is shown in Figure 7.

### 3.4. Ovarian Cancer

**Figure 8:**
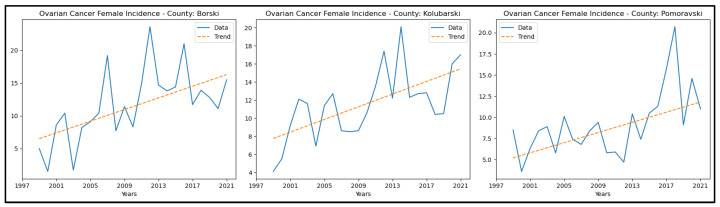
Mann–Kendall analysis of ovarian cancer incidence rates.

The results of the MK analysis of ovarian cancer are presented in Appendix D. It revealed an increasing trend in incidence rates in three counties—Borski, Kolubarski, and Pomoravski—with no significant trend detected in the other counties. The graphical representation of this analysis is shown in Figure 8.

**Figure 9:**
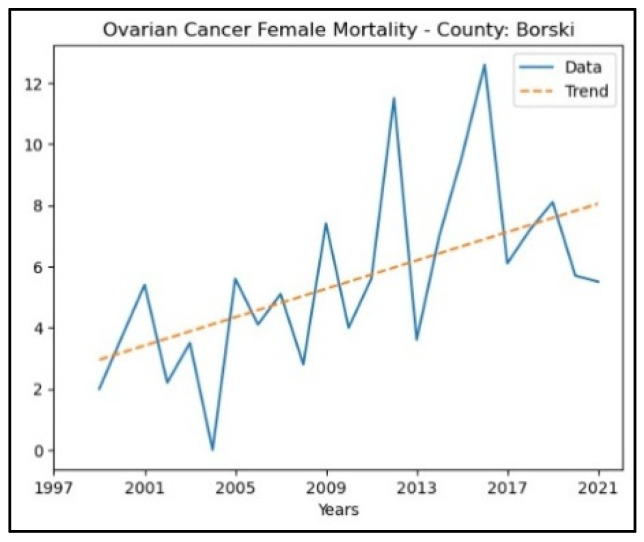
Mann–Kendall analysis of ovarian cancer mortality rates.

The county that recorded an increasing trend in ovarian cancer mortality rates is Borski county, whereas in the other counties, no significant trend was detected. The graphical representation of this analysis is shown in Figure 9.

### 3.5. Uterine Cancer

**Figure 10:**
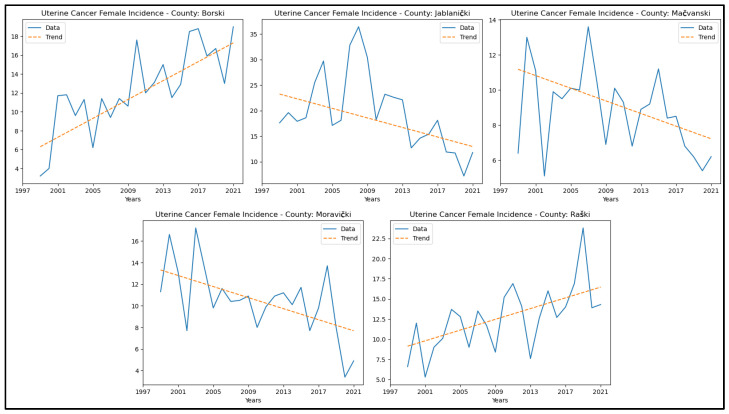
Mann–Kendall analysis of uterine cancer incidence rates.

The results of the MK analysis for uterine cancer are presented in Appendix E. A decreasing trend in uterine cancer incidence rates was identified in two counties: Jablanički and Moravički. An increasing trend in incidence rates for uterine cancer was observed in Borski and Raški counties. No significant trends were detected in the other counties. The graphical representation of this analysis is shown in Figure 10. No study was conducted on the mortality of the specified cancers due to the unavailability of consistent statistical data for the entire analysed period.

### 3.6. Pancreatic Cancer

**Figure 11:**
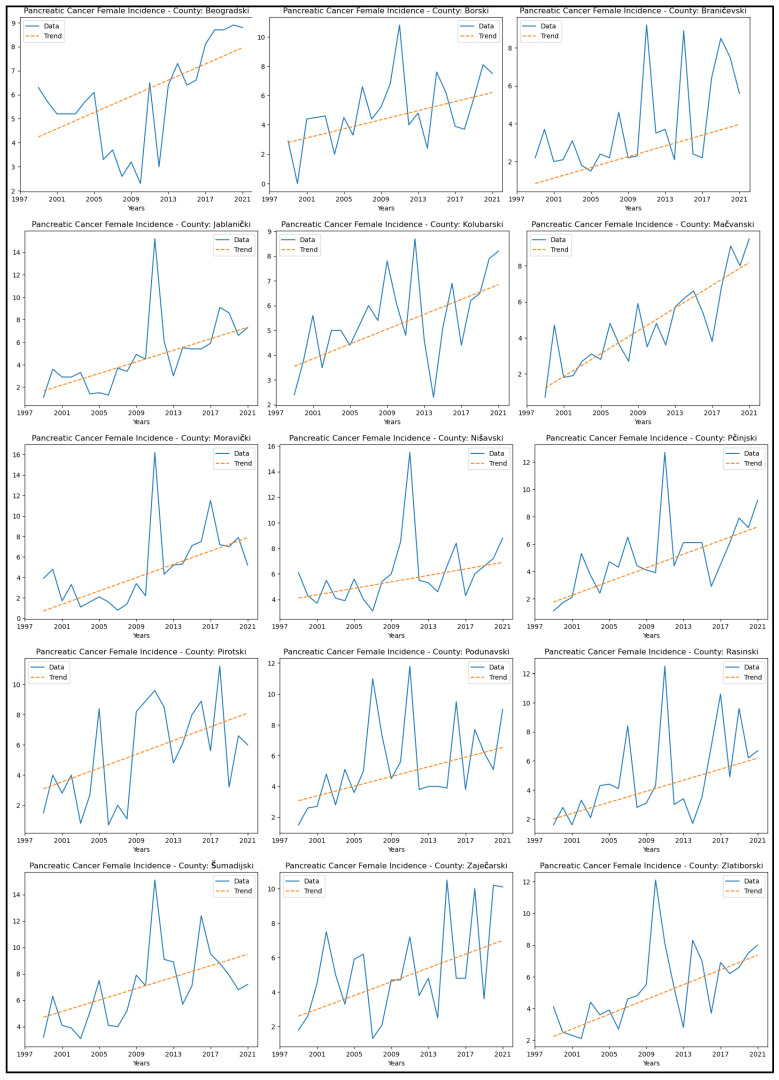
Mann–Kendall analysis of pancreatic cancer incidence rates.

Appendix F presents the parameters from the MK analysis for pancreatic cancer. The graphical representation of this analysis is shown in Figure 11. There is an increasing trend in pancreatic cancer incidence rates in fifteen counties—Beogradski, Borski, Braničevski, Jablanički, Kolubarski, Mačvanski, Moravički, Nišavski, Pčinjski, Pirotski, Podunavski, Rasinski, Šumadijski, Zaječarski, and Zlatiborski.

**Figure 12:**
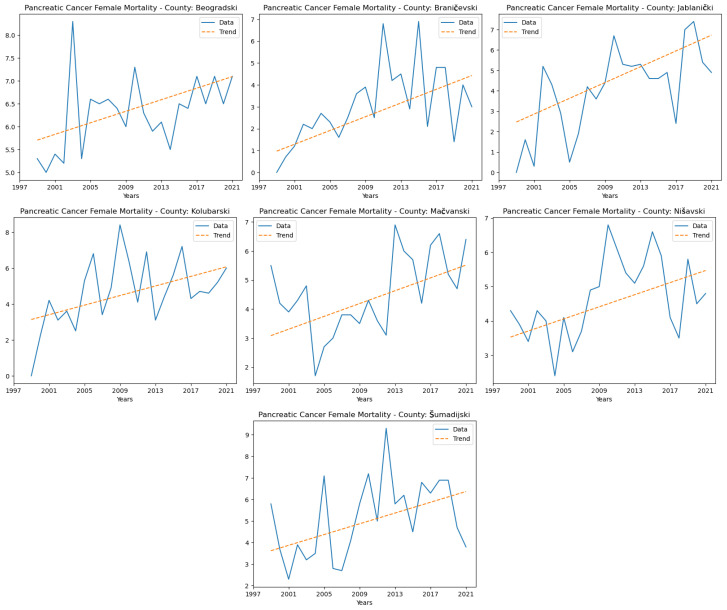
Mann–Kendall analysis of pancreatic cancer mortality rates.

When analysing mortality rate trends for pancreatic cancer, an increasing trend was observed in seven counties: Beogradski, Braničevski, Jablanički, Kolubarski, Mačvanski, Nišavski, and Šumadijski. No significant trends were observed in the other counties. The graphical representation of this analysis is shown in Figure 12.

### 3.7. Bladder Cancer

**Figure 13:**
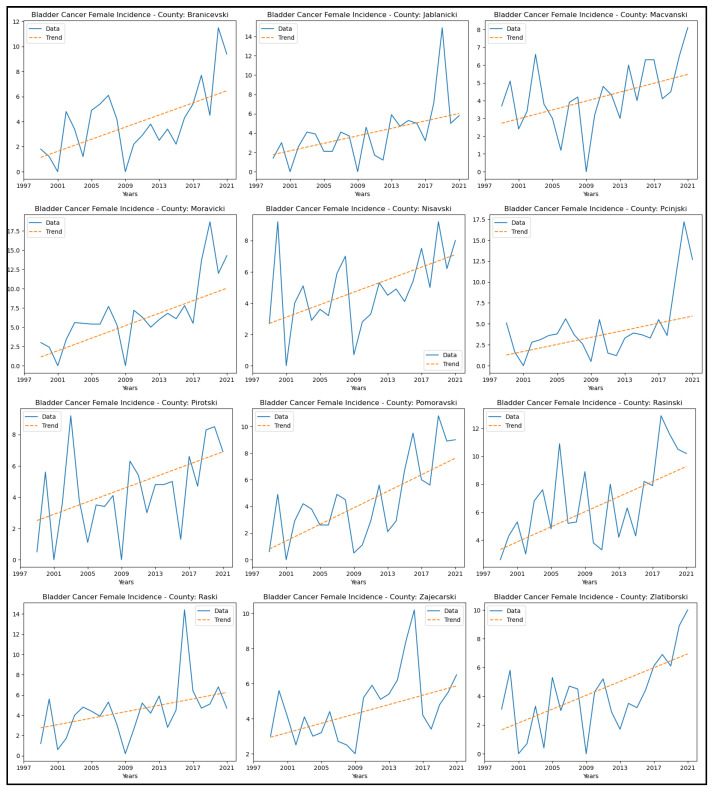
Mann–Kendall analysis of bladder cancer incidence rates.

Appendix G presents the results of this analysis for bladder cancer. Twelve counties showed an upward trend in incidence rates: Braničevski, Jablanički, Mačvanski, Moravički, Nišavski, Pčinjski, Pirotski, Pomoravski, Rasinski, Raški, Zaječarski, and Zlatiborski. No significant trend was observed in the remaining counties. Figure 13 displays the graphical representation of this analysis. No study was conducted on the mortality of the specified cancers due to the unavailability of consistent statistical data for the entire analysed time.

### 3.8. Colorectal Cancer

**Figure 14:**
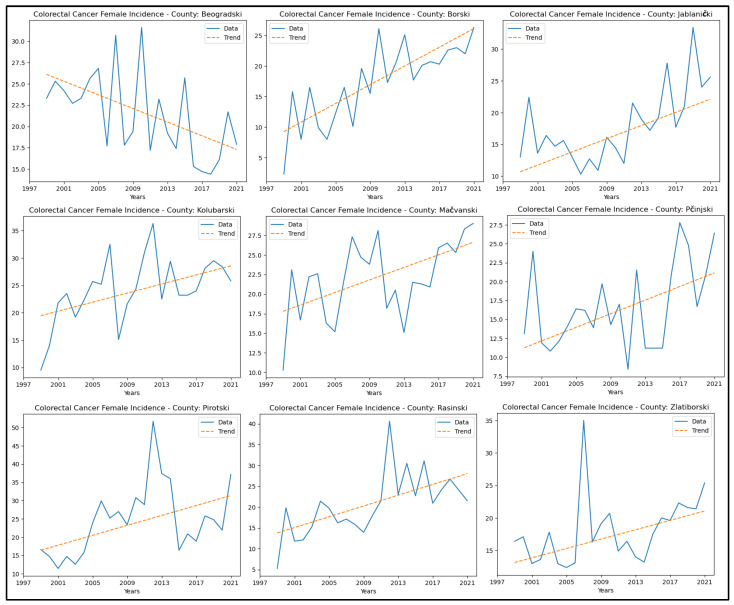
Mann–Kendall analysis of colorectal cancer incidence rates.

Appendix H presents the results of the MK analysis for colorectal cancer. An increasing trend in incidence rates was observed in eight counties: Borski, Jablanički, Kolubarski, Mačvanski, Pčinjski, Pirotski, Rasinski, and Zlatiborski. A decreasing trend in colorectal cancer incidence rates was observed only in Beogradski county. No significant trend was detected in the other counties. The graphical representation of this analysis is shown in Figure 14.

**Figure 15:**
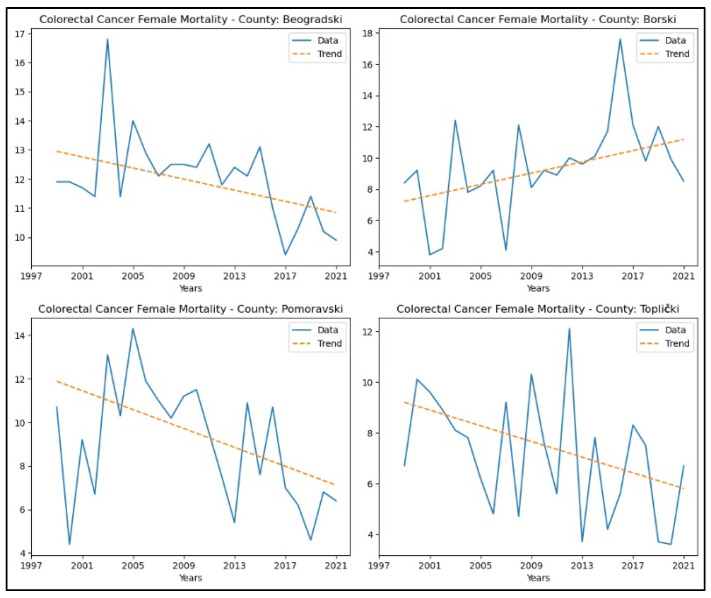
Mann–Kendall analysis of colorectal cancer mortality rates.

An increase in colorectal cancer mortality rates was observed only in Borski county. A decreasing trend in colorectal cancer mortality rates was observed in three counties: Beogradski, Pomoravski, and Toplički. No significant trend in mortality rates for this cancer was detected in the other counties. The graphical representation of this analysis is shown in Figure 15.

### 3.9. Overview of MK Analysis of Cancer Incidence and Mortality Rates by County

As previous graphs showed, regarding cancer incidence rates, it is clear that in Beogradski county, an increasing trend was detected in pancreatic and lung and bronchus cancers, while a decreasing trend was detected in cervical and colorectal cancers. No trends were detected in breast, uterine, bladder, and ovarian cancers. Regarding cancer mortality rates, an increasing trend was detected in pancreatic cancer. A decreasing trend was detected in four cancers: breast, cervical, colorectal, and lung and bronchus. No trend was detected in ovarian cancer.

Borski county. An increasing trend in cancer incidence rates was detected in breast, cervical, uterine, colorectal, ovarian, pancreatic, and lung and bronchus cancers. No trend was detected in two cancers, cervical and bladder. There was no decreasing trend detected in this county. An increasing trend in cancer mortality rates was detected in two cancers: colorectal, and ovarian. A decreasing trend was detected in cervical cancer, while no trend was detected in three cancers: breast, pancreatic and lung and bronchus.

Braničevski county. An increasing trend in cancer incidence rates was detected in three cancers—bladder, pancreatic, and lung and bronchus cancers. A decreasing trend was detected in cervical cancer. No trend was detected for four cancers: breast, uterine, colorectal, and ovarian. Regarding cancer mortality rates, an increasing trend was detected in two cancers: pancreatic and lung and bronchus. There was no decreasing trend detected, while no trend was detected in four cancers: breast, cervical, colorectal, and ovarian.

Jablanički county. In five cancer incidence rates, an increasing trend was detected: breast, colorectal, bladder, pancreatic, and lung and bronchus cancers. A decreasing trend was detected in two cancers, cervical and uterine cancers, while no trend was detected in ovarian cancer. An increasing trend in cancer mortality rates was detected in two cancers: pancreatic and lung and bronchus. There was no decreasing trend detected in this county. No trend was detected in four cancers: breast, cervical, colorectal, and ovarian.

Kolubarski county. An increasing trend in cancer incidence rates was detected in five cancers: breast, colorectal, ovarian, pancreatic, and lung and bronchus cancer. No decreasing trend was detected, while no trend was detected in three cancers: cervical, uterine, and bladder. Regarding cancer mortality rates, an increasing trend was detected in two cancers: breast, and pancreatic. A decreasing trend was not detected, while no trend was detected in four cancers: cervical, colorectal, ovarian, and lung and bronchus cancer.

Mačvanski county. Regarding cancer incidence rates, four increasing trends were detected, in colorectal, bladder, pancreatic, and lung and bronchus cancer. A decreasing trend was detected in uterine cancer. No trend was detected in three cancers: breast, cervical, and ovarian. An increasing trend in cancer mortality rates was detected in pancreatic cancer. There was no decreasing trend detected. No trend was detected in five cancers: breast, cervical, colorectal, ovarian, and lung and bronchus.

Moravički county. An increasing trend in cancer incidence rates was detected in four cancers: breast, bladder, pancreatic, and lung and bronchus. A decreasing trend was detected in cervical and uterine cancers. No trend was detected in colorectal and ovarian cancers. Regarding cancer mortality rates, there was no increasing trend detected in this county. A decreasing trend was detected in two cancers: breast and cervical. No trend was detected in four cancers: colorectal, ovarian, pancreatic, and lung and bronchus.

Nišavski county. An increasing trend in cancer incidence rates was detected in three cancers: bladder, pancreatic, and lung and bronchus cancer. There was no decreasing trend detected, while no trend was detected in five cancers: breast, cervical, uterine, colorectal, and ovarian. An increasing trend in cancer mortality rates was detected in pancreatic cancer. A decreasing trend was not detected in this county, while no trend was detected in five cancers: breast, cervical, colorectal, ovarian, and lung and bronchus.

Pčinjski county. Regarding cancer incidence rates, an increasing trend was detected in five cancers: breast, colorectal, bladder, pancreatic, and lung and bronchus cancer. As well as in previous county, no decreasing trend was detected, while no trend was detected in three cancers: cervical, uterine, and ovarian. Regarding cancer mortality rates, there was no increasing or decreasing trend detected in this county. No trend was detected in all studied cancers.

Pirotski county. An increasing trend in cancer incidence rates was detected in four cancers: colorectal, bladder, pancreatic, and lung and bronchus cancer. There was no decreasing trend detected. No trend was detected in four cancers: breast, cervical, uterine, and ovarian. Regarding cancer mortality rates, as well as in previous county, there was no increasing or decreasing trend detected. No trend was detected in all studied cancers.

Podunavski county. An increasing trend in cancer incidence rates was detected in pancreatic and lung and bronchus cancers. A decreasing trend was detected in cervical cancer, while no trend was detected in five cancers: breast, cervical, colorectal, bladder, and ovarian. A decreasing trend in cancer mortality rates was detected in cervical cancer. There was no increasing trend detected. No trend was detected in all other studied cancers.

Pomoravski county. Regarding cancer incidence rates, an increasing trend was detected in three cancers: bladder, ovarian, and lung and bronchus. There was no decreasing trend detected, while no trend was detected in five cancers: breast, cervical, uterine, colorectal, and pancreatic. A decreasing trend in cancer mortality rates was detected in colorectal cancer, while there was no increasing trend detected. No trend was detected in all other studied cancers.

Rasinski county. An increasing trend in cancer incidence rates was detected in five cancers: breast, colorectal, bladder, pancreatic, and lung and bronchus. There was no decreasing trend detected in this county. No trend was detected for three cancers: cervical, uterine, and ovarian. An increasing trend in cancer mortality rates was detected in lung and bronchus cancers, while no decreasing trend was detected. No trend was detected in all other studied cancers.

Raški county. Regarding cancer incidence rates, an increasing trend was detected in three cancers: uterine, bladder, and lung and bronchus. There was no decreasing trend detected for this county, also. No trend was detected in five cancers: breast, cervical, colorectal, ovarian, and pancreatic. Regarding cancer mortality rates, there was no increasing or decreasing trend detected. No trend was detected in all studied cancers.

Šumadijski county. An increasing trend in cancer incidence rates was detected in two cancers: pancreatic, and lung and bronchus. There was no decreasing trend detected in this county, while no trend was detected in six cancers: breast, cervical, uterine, colorectal, bladder, and ovarian. An increasing trend in cancer mortality rates was detected in pancreatic cancer, while no decreasing trend was detected. No trend was detected in all other studied cancers.

Toplički county. Regarding cancer incidence rates, an increasing trend was detected in lung and bronchus cancers. A decreasing trend was detected in cervical cancer, and no trend was detected in six cancers: breast, uterine, colorectal, bladder, ovarian, and pancreatic. A decreasing trend in cancer mortality rates was detected in colorectal cancer, while there was no increasing trend detected. No trend was detected in all other studied cancers.

Zaječarski county. An increasing trend in cancer incidence rates was detected in three cancers: bladder, pancreatic, and lung and bronchus. A decreasing trend was not detected, while no trend was detected in five cancers: breast, cervical, uterine, colorectal, and ovarian. An increasing trend in cancer mortality rates was detected in lung and bronchus cancer, while no decreasing trend was detected. No trend was detected in all other studied cancers.

Zlatiborski county. Regarding cancer incidence rates, an increasing trend was detected in three cancers: colorectal, bladder, and pancreatic. There was no decreasing trend detected. No trend was detected in five cancers: breast, cervical, uterine, ovarian, and lung and bronchus. Regarding cancer mortality rates, there was no increasing or decreasing trend detected in this county. No trend was detected in all studied cancers.

When comparing trends across counties, certain patterns emerged. Pancreatic cancer most frequently showed increasing incidence rates, often accompanied by upward trends in lung and bronchus cancer. These increases were observed in both urban areas, such as Beogradski county, and in several less densely populated counties, suggesting that the pattern is not confined to a particular type of setting. Conversely, cervical cancer demonstrated the most consistent decreases in incidence, particularly in Beogradski, Jablanički, Moravički, Podunavski, and Toplički counties, likely reflecting the effects of improved screening and prevention measures. Colorectal cancer trends varied considerably, with some counties (e.g., Jablanički, Kolubarski, and Rasinski) showing increases, while others (e.g., Beogradski, Pomoravski, and Toplički) exhibited decreases in either incidence or mortality.

In terms of overall activity, some counties displayed trends in a wide range of cancer types (e.g., Borski and Jablanički, where increases predominated), while others (e.g., Pirotski, Zlatiborski, and Raški) showed few significant changes over the study period. Mortality trends were generally less variable than incidence trends: pancreatic cancer was the most common site of mortality increase, while cervical and colorectal cancers showed the most frequent decreases. These findings suggest that while certain site-specific patterns are consistent across the study area, others are more localised, highlighting the importance of county-level analyses for understanding regional differences in cancer dynamics.

Overall, these results reveal both common and site-specific cancer trends across counties. While certain cancers, such as pancreatic and lung and bronchus, tend to show consistent increases, others, such as cervical and colorectal, more often exhibit decreases in either incidence or mortality.

## 4. Discussion

**Figure 16:**
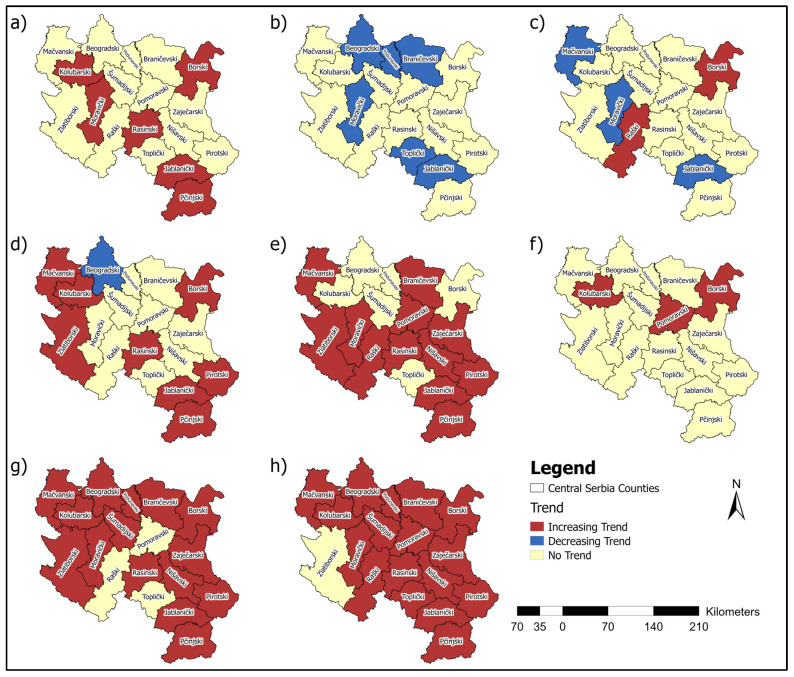
Map of the MK incidence rates analysis for: (**a**) Breast, (**b**) Cervical, (**c**) Uterine, (**d**) Colorectal, (**e**) Bladder, (**f**) Ovarian, (**g**) Pancreatic, and (**h**) Lung and bronchus cancer.

Studies that utilise statistical methods, such as the MK test, hold significant value in analysing temporal trends in the incidence and mortality of malignant diseases. The MK test is a powerful tool for detecting statistically significant changes in time series data. This test enables the identification of long-term and essential trends in disease occurrence, which is crucial for understanding the disease’s dynamics over different periods. MK analysis revealed an increasing incidence of breast cancer in six counties. In contrast, a decreasing trend in cervical cancer incidence and mortality rates was observed in several regions, reflecting possible progress in prevention and early detection. However, increasing trends for colorectal, bladder, pancreatic cancer, and lung and bronchus cancer incidence in multiple counties suggest areas requiring targeted interventions (Figure 16).

Mihalj et al. [54] linked mining activities to an increase in bronchial carcinoma incidence from 2010 to 2020, identifying Borski County as the most affected area. Similarly, our MK analysis confirmed an increasing trend in lung and bronchial cancer cases among women in this county.

According to Sipetić-Grujičić et al. [55], breast cancer was among the most prevalent malignancies affecting women in Central Serbia during the period 1999–2009. Furthermore, the study conducted by Stojanović et al. [52], which analysed the most common gynaecological cancers between 2003 and 2018, revealed a significant upward trend in the incidence of these malignancies across Central Serbia from 2012 to 2018. Specifically, a marked increase in uterine cancer incidence was observed between 2014 and 2018, along with a rise in ovarian cancer cases from 2012 to 2018 [52]. In contrast, cervical cancer showed a slight downward trend in incidence from 2003 to 2015, followed by a marginal increase thereafter [52]. When compared with the findings of our study, we observed an increasing trend in the incidence of uterine cancer in two counties and ovarian cancer in one county. However, since our research spans the extended period from 1999 to 2021 and conducts separate analyses for each of the eighteen counties in Central Serbia, rather than evaluating the region as a whole, direct comparisons with the study above are limited. Nonetheless, both studies indicate a decrease in cervical cancer incidence, which may reflect the positive impact of prevention programmes and early detection efforts implemented in this region.

Mortality trends indicated regional variations, with increasing rates for lung and bronchus cancer county. Additionally, a study conducted by Ilić, M., and Ilić, I. [44,45] analysed cancer mortality from 1999 to 2015, revealing a notable increase in lung cancer mortality in four counties (Braničevski, Jablanički, Rasinski, and Zaječarski) and decreasing rates in Beogradski among females.

Marković-Denić et al. [95] reported increasing mortality trends for both sexes in Central Serbia between 1985 and 2002, particularly for lung and colorectal cancers. Our MK analysis, covering a more recent period, detected increased lung cancer mortality in four counties and colorectal cancer mortality in one county, differences likely reflecting improvements in public health policies, cancer prevention, and awareness over time.

Kričković et al. [47] conducted a spatiotemporal and trend analysis of cancer incidence among men in Central Serbia. In [47] found rising incidence rates among men in several cancer types, with the broadest spatial increases for colorectal and pancreatic cancers. In contrast, our analysis of the female population revealed a wider distribution of increased incidence for lung and bronchus, bladder, and pancreatic cancers. Mortality trends also differed by sex, with colorectal cancer mortality more prevalent in men and pancreatic cancer mortality more widespread among women. For lung and bronchus cancer mortality, both studies identified the same counties with persistent increases or decreases, indicating stable geographic patterns over time. These results highlight the need for sex-specific cancer surveillance and sustained, regionally tailored public health interventions to address persistent high-mortality areas.

**Figure 17:**
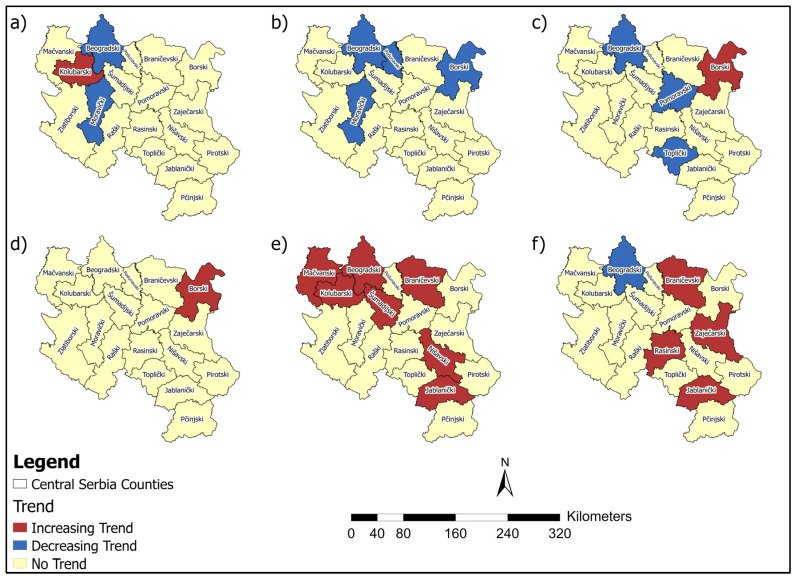
Map of the MK mortality rates analysis for (**a**) Breast, (**b**) Cervical, (**c**) Colorectal, (**d**) Ovarian, (**e**) Pancreatic and (**f**) Lung and bronchus cancer.

Pancreatic cancer showed increasing mortality rates in seven counties, which also represents a significant public health concern. Decreasing mortality rates for breast, cervical, and colorectal cancers in certain counties highlight potential advances in treatment and healthcare access (Figure 17). These findings underscore the need for tailored public health strategies, emphasising prevention, early diagnosis, and resource allocation to address both the rising and declining trends in cancer incidence and mortality across Central Serbia.

Since the Mann–Kendall test is independent of data distribution, it is particularly suitable for analysing data with non-specific distributions. This method allows for accurate analysis even when incidence and mortality data are imperfect or incomplete. As a result, it has broad applicability in regions where data quality is often limited, such as in many developing countries, including Serbia. This analysis is focused on a single statistical method due to the large study area and the unavailability of data regarding public health concerning cancers and emergency response.

One of the key limitations of this study is the unavailability of consistent and reliable mortality data for certain types of cancer, which restricts the application of comprehensive spatial and temporal analyses. The absence of such data reduces the ability to fully assess the geographical and temporal dynamics of cancer burden, thereby limiting the generalizability of the findings. Additionally, the lack of access to disaggregated data at the municipal and settlement levels impedes the identification of micro-regional disparities, which are essential for understanding local variations in cancer risk and outcomes. These limitations highlight the need for more granular data collection and reporting mechanisms, particularly at the local administrative levels.

Future research should prioritise small-area analysis to detect spatial clusters and emerging trends that are otherwise obscured at broader spatial scales. Such approaches would provide a foundation for targeted public health interventions, allowing for a more precise allocation of resources to areas with the greatest need [96].

Furthermore, this study highlights the importance of integrating clinical and demographic variables, such as tumour staging, treatment regimens, and patient outcomes, into spatial epidemiological analyses. The inclusion of such variables could significantly enhance the explanatory power of spatial patterns and improve our understanding of the multifactorial nature of cancer incidence and mortality [96].

The increasing prevalence of chronic and non-communicable diseases, including cancer, poses a significant yet often overlooked challenge for disaster and public health preparedness [97,98,99,100,101]. This study indicates that in Central Serbia, female-specific cancers exhibit spatial and temporal trends that could worsen existing vulnerabilities during crises, particularly in areas with inadequate healthcare resources and heightened environmental exposure risks.

Air pollution is a major public health concern in Serbia, ranked fifth among 37 European countries in IQAir’s 2019 World Air Quality Report, with a population-adjusted annual PM2.5 mean of 23.3 μg/m^3^ [47,102]. Main sources include lignite- and coal-fired power plants, coal and wood heating, and emissions from outdated vehicles, industry, waste sites, and agriculture [47,102,103]. While global evidence strongly links air pollution to cancer, such studies are scarce for Central Serbia, highlighting the need for further research.

Other established cancer risk factors include smoking, alcohol use, radiation, toxic substances, Epstein–Barr virus, pernicious anaemia, gastroesophageal reflux, blood type A, low socioeconomic status, and genetic predisposition [47,104,105,106]. Smoking alone is causally linked to multiple cancers, while combined with alcohol, it markedly increases the risk of cancers of the oesophagus, oral cavity, and pharynx [47,107,108,109]. Reducing tobacco and alcohol use remains a public health priority in Central Serbia. Despite existing tobacco control laws, stronger multi-sectorial strategies are needed. The WHO ranks alcohol use among the top 10 global disease burden risk factors [47,110].

A 2019 national health survey revealed pronounced regional disparities: Southern and Eastern Serbia showed the highest obesity, daily bread intake, youth smoking, and alcohol use, while Šumadija and Western Serbia had the lowest daily smoking and alcohol consumption [47,111]. These findings underline the importance of region-specific interventions to reduce exposure to modifiable risk factors. These lifestyle disparities align with the regional patterns of cancer incidence and mortality identified in our study, underscoring the influence of behavioural and socioeconomic factors on cancer risk. Addressing these underlying determinants is crucial for tailoring effective public health interventions and integrating cancer control into disaster preparedness frameworks, ultimately enhancing the resilience of healthcare systems across Central Serbia.

Regions with consistently rising cancer rates may experience increased stress during natural hazards, pandemics, or technological accidents, arising from diminished population resilience, greater healthcare demands, and slower emergency responses [112,113,114,115,116]. Women with untreated or advanced-stage cancers, particularly in rural or underserved communities, face an elevated risk of interrupted care during emergencies, which exacerbates existing health inequalities [117,118,119,120].

In this context, incorporating cancer surveillance and spatial health analysis into national risk assessments and early warning systems is essential. Identifying clusters of chronic disease burden can aid in prioritising resources, enhancing community-level preparedness planning, and strengthening overall public health resilience [4,121,122]. This integrated approach is particularly crucial in low- and middle-income regions, where disaster response capacities and chronic disease management often fall behind.

The results also suggest a need for more extensive fieldwork and epidemiological surveillance in areas that have shown consistently high or increasing cancer rates. These regions warrant special attention for investigating potential environmental exposures, lifestyle-related risk factors, and socio-economic determinants. Incorporating interdisciplinary methodologies, combining geospatial analysis with clinical and public health data, would strengthen future studies and contribute to evidence-based decision-making.

Despite these scientific imperatives, limited financial resources and infrastructural capacities persist as significant barriers to the implementation of advanced cancer research in Serbia. Strengthening institutional frameworks, improving data infrastructure, and fostering international collaborations will be crucial for overcoming these challenges and ensuring the sustainability of cancer surveillance and control efforts.

Studies that investigate trends in the incidence and mortality of malignant diseases play a vital role in the modern approach to public health planning and disease prevention [123,124,125,126,127,128]. By identifying trends, timely detection of areas with increased risk is made possible, which forms the basis for efficiently targeting interventions, allocating resources, and implementing preventive measures [128,129,130,131,132]. In the context of the increasing burden on healthcare systems due to the rising number of cancer cases, such research enables data-driven decision-making, thus enhancing the effectiveness of healthcare policies and improving access to diagnosis and treatment in the most vulnerable communities [133,134,135,136].

The recommendations provided in Table 2 are directly informed by the spatial–temporal trends in cancer incidence and mortality detected between 1999 and 2021. For each cancer type, actions were prioritised in regions where statistically significant increasing trends were observed (either incidence, mortality, or both), under the assumption that sustained increases over multiple years signal gaps in prevention, screening, diagnosis, or treatment. Conversely, areas with no or decreasing trends were considered potential models for good practice transfer. While temporal trends provide a valuable early-warning signal for regional disparities, they cannot alone define a complete intervention strategy. Ideally, such recommendations should be refined by integrating demographic, socioeconomic, behavioural, and health-system variables. In the absence of these data, the present recommendations are framed as preliminary, evidence-informed measures that can guide more detailed, multi-factorial planning.

Linking these findings with emergency preparedness is critical for effective policy action. Increasing trend cancer regions should be prioritised in disaster response planning, with measures such as maintaining uninterrupted oncology services, securing treatment supply chains, and deploying mobile care units during crises. Integrating cancer surveillance into national preparedness frameworks would allow timely, evidence-based resource allocation, reduce treatment disruptions, and strengthen overall health-system resilience in the face of natural hazards, pandemics, or other emergencies.

## 5. Conclusions

As shown in this study, cancers are highly prevalent among the female population in Central Serbia. This is the first study in Central Serbia to apply the Mann–Kendall test to analyse cancer trends among the female population from 1999 to 2021. Additionally, it is one of the pioneering geographic-medical studies in the region that examines cancer in the female population.

Despite their advantages, the Mann–Kendall test and Sen’s slope estimator have notable limitations. Both methods assume monotonic trends and may fail to detect non-linear, cyclical, or abrupt changes in cancer incidence, which can arise from sudden policy changes, environmental exposures, or advances in screening technologies. They are also sensitive to strong serial correlation in the data, which, if unaddressed, can increase the likelihood of falsely identifying a trend. Furthermore, these methods evaluate temporal patterns independently of spatial autocorrelation, meaning they do not account for the possibility that neighbouring regions may exhibit similar cancer trends due to shared risk factors. For a comprehensive geospatial analysis of cancer patterns, the Mann–Kendall test and Sen’s slope are most effective when used in conjunction with spatial statistical techniques and models that can accommodate non-linearities and spatial dependencies.

Research of this type requires interdisciplinary collaboration among oncologists, geographers, epidemiologists, and experts in information technology. The use of GIS tools and statistical methods enables a detailed analysis of complex patterns that would remain invisible without such an approach. In this way, not only is a better understanding of the spatial aspects of health achieved, but also the foundations are laid for future research and the improvement of public health at both national and local levels. For this reason, this type of research represents a crucial step toward developing more precise, equitable, and sustainable strategies for controlling and preventing malignant diseases, especially in developing countries where healthcare resources are limited and spatial inequalities are often pronounced. The research was conducted according to the STROBE statement (Supplementary Materials).

Additionally, this study highlights the importance of integrating cancer surveillance into broader disaster risk reduction and public health resilience strategies. As chronic diseases increasingly influence the health vulnerabilities faced by populations, particularly women, their inclusion in national preparedness and adaptation plans becomes essential. The increasing incidence of cancer, especially lung, pancreatic, and colorectal cancers, highlights the necessity for improved screening, environmental monitoring, and access to early diagnostics in areas with high risk.

To effectively harness the promise of geospatial epidemiology, future research must integrate more detailed spatial data, examine relationships with environmental and socioeconomic factors, and include clinical information such as tumour staging and treatment outcomes. Enhancing data systems, promoting interdisciplinary collaboration, and securing ongoing institutional and financial backing will be essential for converting research insights into successful cancer control strategies. Furthermore, this study lays the groundwork for understanding cancer trends in Central Serbia and creates opportunities for more targeted, equitable, and informed interventions. It is crucial to view cancer as a multifaceted challenge, encompassing medical, spatial, and social aspects, to achieve lasting health security and resilience.

From a scientific perspective, this study demonstrates the value of applying non-parametric statistical methods and geospatial technologies to long-term epidemiological data, providing a replicable framework for analysing cancer trends in other regions with similar data limitations. It contributes to the expanding field of spatial epidemiology by bridging gaps between medical research, environmental science, and public health planning. On the other side, from a societal perspective, the findings demand immediate attention from policymakers and public health officials to tackle cancer not just as a medical issue, but also as an environmental and structural dilemma. These insights can help allocate resources, direct local interventions, and inform awareness initiatives focused on early detection and prevention, particularly for vulnerable populations such as women in underserved areas. Ensuring equitable access to healthcare, diagnostics, and preventive services is crucial for reducing geographic health disparities and strengthening the resilience of health systems against chronic disease challenges.

## Figures and Tables

**Table 1:** Demographic characteristics.

County/Region/Area	Population Number			Percentage in Women	Average Age		
	Total	Men	Women		Total	Men	Women
Beogradski	1,681,405	794,413	886,992	52.8%	42.7	41.0	44.3
Borski	101,100	49,453	51,647	51.1%	47.5	46.0	49.0
Braničevski	156,367	76,881	79,486	50.8%	46.5	44.9	48.1
Jablanički	184,502	92,220	92,282	50.0%	44.4	43.5	45.3
Kolubarski	154,497	76,563	77,934	50.4%	45.0	43.8	46.1
Mačvanski	265,377	131,188	134,189	50.6%	44.4	43.3	45.5
Moravički	189,281	92,817	96,464	51.0%	45.1	43.7	46.4
Nišavski	343,950	168,367	175,583	51.0%	44.3	43.2	45.4
Pčinjski	193,802	97,273	96,529	49.8%	41.7	41.0	42.5
Pirotski	76,700	38,762	37,938	49.5%	47.2	46.4	47.9
Podunavski	175,573	86,811	88,762	50.6%	44.0	42.5	45.5
Pomoravski	182,047	88,333	93,714	51.5%	46.2	44.8	47.6
Rasinski	207,197	101,873	105,324	50.8%	46.0	44.6	47.2
Raški	296,532	147,411	149,121	50.3%	40.7	39.6	41.7
Šumadijski	269,728	131,388	138,340	51.3%	44.2	42.9	45.3
Toplički	77,341	39,031	38,310	49.5%	44.3	43.4	45.2
Zaječarski	96,715	47,188	49,527	51.2%	48.6	47.0	50.2
Zlatiborski	254,659	126,267	128,392	50.4%	44.9	43.8	46.0
Serbia—South	3,225,368	1,591,826	1,633,542	50.6%	44.6	43.3	45.7
Region of Šumadija and Western Serbia	1,819,318	895,840	923,478	50.8%	44.3	43.1	45.5
Region of Southern and Eastern Serbia	1,406,050	695,986	710,064	50.5%	44.9	43.7	46.0
Serbia	6,647,003	3,231,978	3,415,025	51.4%	43.9	42.4	45.2

Source: Ref. [93], adjusted.

**Table 2:** Regional cancer trends among women in Central Serbia (1999–2021) and corresponding public health and disaster preparedness recommendations.

Research Focus Area	Detected Trends (1999–2021)	Recommended Actions
Breast cancer incidence rates (6 counties)	Increasing trends in Borski, Jablanički, Kolubarski, Moravički, Pčinjski, Rasinski	Strengthen local screening programmes (mammography), deploy mobile units; educate health workers and the public; integrate oncology protocols into crisis plans.
Breast cancer mortality rates	Increase in Kolubarski; decreases in Beogradski and Moravički	Evaluate treatment accessibility and transportation in Kolubarski; share best practices from regions with declining mortality; implement rapid response teams for treatment continuity.
Cervical cancer incidence and mortality rates	Declines in Beogradski, Braničevski, Jablanički, Moravički, Podunavski, Toplički (incidence and/or mortality)	Expand HPV vaccination; update and broaden screening coverage; monitor areas where decline is not observed.
Lung/bronchus cancer incidence and mortality rates	Incidence up in 17 counties; mortality up in 16	Broaden anti-smoking initiatives; adopt early diagnostic protocols; integrate oncology scenarios into disaster and pollution response plans.
Ovarian cancer incidence and mortality rates	Incidence up in Borski, Kolubarski, Pomoravski; mortality up in Borski	Launch reproductive health education programmes; support gynecologic oncology clinics in affected regions; include protocols in emergency preparedness plans.
Uterine cancer incidence rates	Increase in Borski and Raški; decrease in Jablanički and Moravički	Investigate causes of regional variation; apply targeted public health strategies (education, early testing).
Pancreatic cancer incidence and mortality rates	Incidence up in 15 counties; mortality up in 7	Develop early diagnostic approaches (biomarkers, imaging); train medical staff in symptom recognition; include oncology in mobile crisis health units.
Bladder cancer incidence rates	Increase in 12 counties	Monitor exposure factors (pollution, occupational risks); promote healthy diets and risk reduction; include as long-term health risks in crisis scenarios.
Colorectal cancer incidence and mortality rates	Increased incidence in 8 counties; only Borski saw increased mortality rates	Promote dietary interventions and regular screenings (e.g., colonoscopy); analyse regional health trends; prioritise outpatient clinics in emergency planning.

## Data Availability

Data are contained within the article.

## Supplementary Materials

The following supporting information can be downloaded at: https://www.mdpi.com/article/10.3390/healthcare13172169/s1, Table S1. STROBE Statement.

## Appendix A

Parameters of the MK test for breast cancer at the significance level of 0.05 by county from 1999 to 2021 in the study area (*p*—*p*-value of the significance test, Z—standardised test statistics, s—so-called Sen’s slope, b—intercept).

**County**	**Breast Cancer**							
	**Incidence**				**Mortality**			
	***p***	**Z**	**s**	**b**	***p***	**Z**	**s**	**b**
Beogradski	1.00	0	0	74.50	1.03 × 10^−3^	−3.28	−0.17	25.59
Borski	0.01	2.59	1.25	43.55	0.07	−1.80	−0.21	20.94
Braničevski	0.65	−0.45	−0.13	44.88	0.26	1.14	0.05	15.25
Jablanički	2.18 × 10^−4^	3.70	1.50	30.90	0.09	1.72	0.20	15.10
Kolubarski	1.95 × 10^−4^	3.73	1.13	41.70	0.01	2.80	0.29	14.12
Mačvanski	0.09	1.72	0.51	44.36	0.71	0.37	0.04	17.29
Moravički	9.56 × 10^−8^	5.33	1.40	47.60	0.02	−2.25	−0.24	22.04
Nišavski	0.12	−1.56	−0.31	69.77	0.98	−0.03	−0.02	20.27
Pčinjski	1.04 × 10^−3^	3.28	1.41	27.12	0.65	0.45	0.05	15.11
Pirotski	0.22	1.21	0.40	53.80	0.40	−0.85	−0.08	19.18
Podunavski	0.87	0.16	0.06	54.47	0.69	−0.40	−0.04	20.88
Pomoravski	0.09	1.72	0.76	43.86	0.24	−1.16	−0.12	18.55
Rasinski	0.03	2.22	1.10	60.90	0.60	−0.53	−0.05	20.70
Raški	0.05	1.93	0.55	50.21	0.23	1.19	0.10	19.00
Šumadijski	0.05	1.93	0.34	68.16	0.83	−0.21	−0.02	19.18
Toplički	0.79	0.26	0.08	47.48	1.00	0	0	14.30
Zaječarski	0.37	0.90	0.19	44.29	0.27	−1.11	−0.12	18.67
Zlatiborski	0.69	−0.40	−0.09	52.69	0.58	0.56	0.06	16.44

## Appendix B

Parameters of the MK test for cervical cancer at the significance level of 0.05 by county from 1999 to 2021 in the study area (*p*-value of the significance test, Z-standardised test statistics, s-so-called Sen’s slope, b-intercept).

**County**	**Cervical Cancer**							
	**Incidence**				**Mortality**			
	***p***	**Z**	**s**	**b**	***p***	**Z**	**s**	**b**
Beogradski	2.65 × 10^−4^	−3.65	−0.62	28.62	0.02	−2.39	−0.06	6.83
Borski	0.96	−0.05	−0.02	34.78	0.01	−2.59	−0.29	13.74
Braničevski	0.02	−2.32	−0.89	29.19	0.13	−1.51	−0.11	9.61
Jablanički	0.01	−2.48	−0.41	26.18	0.58	−0.56	−0.03	6.25
Kolubarski	0.18	1.35	0.24	18.79	0.94	−0.08	0	6.60
Mačvanski	0.19	−1.30	−0.11	17.78	0.56	0.58	0.03	6.73
Moravički	0.01	−2.80	−0.59	32.53	0.01	−2.75	−0.22	10.42
Nišavski	0.62	−0.50	−0.06	23.01	0.94	−0.08	0	7.00
Pčinjski	0.75	−0.32	−0.12	24.08	0.56	0.58	0.05	6.25
Pirotski	0.06	1.90	0.43	15.43	0.63	0.48	0.05	3.71
Podunavski	0.01	−2.48	−0.36	24.03	0.01	−2.59	−0.32	11.89
Pomoravski	0.34	−0.95	−0.15	19.85	0.87	−0.16	−0.03	8.29
Rasinski	0.12	−1.56	−0.22	20.68	0.41	0.82	0.04	5.12
Raški	0.07	−1.80	−0.38	23.95	0.56	−0.58	−0.03	8.08
Šumadijski	0.09	−1.72	−0.34	28.84	0.34	−0.95	−0.08	7.66
Toplički	0.04	−2.06	−0.42	25.77	0.27	1.11	0.11	5.74
Zaječarski	0.37	−0.90	−0.23	29.37	0.71	−0.37	−0.07	9.33
Zlatiborski	0.30	−1.03	−0.17	19.79	0.81	−0.24	−0.01	6.96

## Appendix C

Parameters of the MK test for the lung and bronchus cancer at the significance level of 0.05 by county from 1999 to 2021 in the study area (*p*-value of the significance test, Z-standardised test statistics, s-so-called Sen’s slope, b-intercept).

**County**	**Lung and Bronchus Cancer**							
	**Incidence**				**Mortality**			
	***p***	**Z**	**s**	**b**	***p***	**Z**	**s**	**b**
Beogradski	0.02	2.43	0.44	19.96	4.64 × 10^−5^	4.07	0.35	17.55
Borski	4.17 × 10^−5^	4.10	0.70	9.00	1.39 × 10^−3^	3.20	0.53	6.13
Braničevski	7.09 × 10^−4^	3.39	0.50	10.50	3.98 × 10^−4^	3.54	0.40	8.90
Jablanički	1.26 × 10^−3^	3.22	0.58	6.41	3.62 × 10^−4^	3.57	0.42	6.00
Kolubarski	1.42 × 10^−5^	4.34	0.59	10.71	0.03	2.17	0.26	10.15
Mačvanski	1.74 × 10^−6^	4.78	0.72	9.58	1.96 × 10^−6^	4.76	0.44	10.25
Moravički	4.25 × 10^−6^	4.60	0.36	14.04	0.03	2.17	0.22	9.82
Nišavski	2.40 × 10^−4^	3.67	0.46	10.29	9.36 × 10^−8^	5.34	0.51	6.73
Pčinjski	7.25 × 10^−5^	3.97	0.98	3.12	0.13	1.53	0.24	9.73
Pirotski	0.02	2.30	0.27	8.47	0.75	0.32	0.03	8.76
Podunavski	1.15 × 10^−4^	3.86	0.94	12.53	2.95 × 10^−4^	3.62	0.57	14.41
Pomoravski	0.01	2.77	0.59	6.40	0.04	2.01	0.20	13.10
Rasinski	0.01	2.67	0.53	12.53	1.75 × 10^−4^	3.75	0.33	7.09
Raški	3.36 × 10^−3^	2.93	0.41	9.64	2.17 × 10^−3^	3.07	0.34	7.80
Šumadijski	3.95 × 10^−3^	2.88	0.29	17.26	4.22 × 10^−5^	4.10	0.50	9.70
Toplički	0.01	2.64	0.42	10.48	0.01	2.67	0.39	6.26
Zaječarski	1.02 × 10^−5^	4.41	0.78	6.78	1.05 × 10^−3^	3.28	0.44	5.25
Zlatiborski	0.17	1.37	0.23	11.53	0.01	2.78	0.28	8.08

## Appendix D

Parameters of the MK test for ovarian cancer at the significance level of 0.05 by county from 1999 to 2021 in the study area (*p*-value of the significance test, Z-standardised test statistics, s-so-called Sen’s slope, b-intercept).

**County**	**Ovarian Cancer**							
	**Incidence**				**Mortality**			
	***p***	**Z**	**s**	**b**	***p***	**Z**	**s**	**b**
Beogradski	0.07	−1.80	−0.10	12.50	0.83	0.21	0.01	5.80
Borski	2.38 × 10^−3^	3.04	0.44	6.51	2.38 × 10^−3^	3.04	0.23	2.95
Braničevski	0.41	0.82	0.16	6.59	0.44	0.77	0.07	3.03
Jablanički	0.09	1.69	0.21	6.94	0.19	1.32	0.08	3.88
Kolubarski	2.17 × 10^−3^	3.07	0.35	7.75	0.18	1.35	0.07	3.77
Mačvanski	0.18	1.35	0.07	7.73	0.65	0.45	0.02	3.86
Moravički	0.18	1.35	0.13	8.39	0.21	−1.24	−0.10	6.65
Nišavski	0.96	−0.05	−0.01	12.98	0.23	1.19	0.04	4.73
Pčinjski	0.07	1.80	0.23	5.93	0.83	0.21	0.02	3.80
Pirotski	0.10	1.64	0.20	8.80	0.92	0.11	0.01	3.39
Podunavski	0.71	0.37	0.05	9.52	0.19	−1.30	−0.05	5.21
Pomoravski	3.95 × 10^−3^	2.88	0.30	5.20	0.29	1.06	0.06	4.19
Rasinski	0.98	−0.03	0.00	9.70	0.09	1.72	0.10	3.30
Raški	0.58	0.55	0.06	9.11	0.81	−0.24	−0.02	5.08
Šumadijski	0.15	1.45	0.15	10.85	0.12	−1.54	−0.06	5.78
Toplički	0.96	0.05	0.02	11.60	0.53	−0.63	−0.03	3.37
Zaječarski	0.62	0.50	0.05	9.25	0.49	−0.69	−0.05	6.45
Zlatiborski	0.20	1.27	0.16	8.59	0.10	1.67	0.07	3.90

## Appendix E

Parameters of the MK test for the uterine cancer at the significance level of 0.05 by county from 1999 to 2021 in the study area (*p*—*p*-value of the significance test, Z—standardised test statistics, s—so-called Sen’s slope, b—intercept).

**County**	**Uterine Cancer**							
	**Incidence**				**Mortality**			
	***p***	**Z**	**s**	**b**	***p***	**Z**	**s**	**b**
Beogradski	0.65	−0.45	−0.07	14.65	N/A	N/A	N/A	N/A
Borski	3.36 × 10^−5^	4.15	0.50	6.30	N/A	N/A	N/A	N/A
Braničevski	0.40	−0.85	−0.12	12.15	N/A	N/A	N/A	N/A
Jablanički	0.01	−2.56	−0.47	23.23	N/A	N/A	N/A	N/A
Kolubarski	0.33	0.98	0.10	10.30	N/A	N/A	N/A	N/A
Mačvanski	0.02	−2.41	−0.18	11.18	N/A	N/A	N/A	N/A
Moravički	0.02	−2.38	−0.26	13.31	N/A	N/A	N/A	N/A
Nišavski	0.51	0.66	0.08	16.58	N/A	N/A	N/A	N/A
Pčinjski	0.41	0.82	0.10	14.80	N/A	N/A	N/A	N/A
Pirotski	0.81	0.24	0.01	12.32	N/A	N/A	N/A	N/A
Podunavski	0.27	−1.11	−0.08	10.87	N/A	N/A	N/A	N/A
Pomoravski	0.75	0.32	0.03	8.99	N/A	N/A	N/A	N/A
Rasinski	0.85	−0.19	−0.02	17.40	N/A	N/A	N/A	N/A
Raški	1.82 × 10^−3^	3.12	0.33	9.13	N/A	N/A	N/A	N/A
Šumadijski	0.62	−0.50	−0.05	13.15	N/A	N/A	N/A	N/A
Toplički	0.25	1.16	0.14	9.76	N/A	N/A	N/A	N/A
Zaječarski	0.21	1.24	0.18	9.48	N/A	N/A	N/A	N/A
Zlatiborski	0.20	−1.27	−0.11	9.54	N/A	N/A	N/A	N/A

## Appendix F

Parameters of the MK test for the pancreatic cancer at the significance level of 0.05 by county from 1999 to 2021 in the study area (*p*—*p*-value of the significance test, Z—standardised test statistics, s—so-called Sen’s slope, b—intercept).

**County**	**Pancreatic Cancer**							
	**Incidence**				**Mortality**			
	***p***	**Z**	**s**	**b**	***p***	**Z**	**s**	**b**
Beogradski	3.59 × 10^−3^	2.91	0.17	4.24	0.02	2.28	0.06	5.71
Borski	0.02	2.27	0.16	2.80	0.28	−1.08	−0.06	5.05
Braničevski	0.01	2.47	0.14	0.84	1.52 × 10^−3^	3.17	0.16	0.97
Jablanički	4.70 × 10^−5^	4.07	0.26	1.67	7.11 × 10^−4^	3.39	0.19	2.47
Kolubarski	0.01	2.70	0.15	3.55	0.01	2.46	0.13	3.13
Mačvanski	2.88 × 10^−6^	4.68	0.32	1.22	0.04	2.09	0.11	3.08
Moravički	1.82 × 10^−3^	3.12	0.33	0.73	0.21	1.24	0.07	3.77
Nišavski	0.02	2.38	0.13	4.11	0.04	2.06	0.09	3.53
Pčinjski	4.24 × 10^−4^	3.52	0.25	1.75	0.65	0.45	0.01	3.74
Pirotski	0.03	2.11	0.23	3.09	0.46	0.74	0.07	4.21
Podunavski	0.01	2.46	0.16	3.07	0.58	0.56	0.03	4.13
Pomoravski	0.07	1.82	0.13	1.93	0.62	−0.50	−0.03	5.59
Rasinski	3.34 × 10^−3^	2.93	0.19	2.01	0.13	−1.51	−0.07	5.69
Raški	0.60	0.53	0.05	5.05	0.43	0.79	0.07	4.65
Šumadijski	3.34 × 10^−3^	2.93	0.22	4.72	0.04	2.06	0.13	3.63
Toplički	0.77	0.29	0.03	4.13	0.40	−0.85	−0.11	5.41
Zaječarski	0.04	2.06	0.20	2.60	0.06	1.85	0.12	3.20
Zlatiborski	0.00	3.33	0.23	2.23	0.15	1.45	0.07	3.53

## Appendix G

Parameters of the MK test for the bladder cancer at the significance level of 0.05 by county from 1999 to 2021 in the study area (*p*-value of the significance test, Z-standardised test statistics, s-so-called Sen’s slope, b-intercept).

**County**	**Bladder Cancer**							
	**Incidence**				**Mortality**			
	***p***	**Z**	**s**	**b**	***p***	**Z**	**s**	**b**
Beogradski	0.73	0.34	0.03	4.83	N/A	N/A	N/A	N/A
Borski	0.83	−0.21	−0.03	5.33	N/A	N/A	N/A	N/A
Braničevski	0.01	2.67	0.24	1.14	N/A	N/A	N/A	N/A
Jablanički	1.51 × 10^−3^	3.17	0.19	1.77	N/A	N/A	N/A	N/A
Kolubarski	0.20	1.27	0.10	3.30	N/A	N/A	N/A	N/A
Mačvanski	0.01	2.48	0.13	2.73	N/A	N/A	N/A	N/A
Moravički	1.56 × 10^−4^	3.78	0.41	1.13	N/A	N/A	N/A	N/A
Nišavski	0.01	2.62	0.20	2.70	N/A	N/A	N/A	N/A
Pčinjski	0.02	2.33	0.21	1.27	N/A	N/A	N/A	N/A
Pirotski	0.02	2.33	0.20	2.50	N/A	N/A	N/A	N/A
Podunavski	0.07	1.82	0.16	3.91	N/A	N/A	N/A	N/A
Pomoravski	1.49 × 10^−3^	3.18	0.31	0.78	N/A	N/A	N/A	N/A
Rasinski	0.01	2.75	0.27	3.33	N/A	N/A	N/A	N/A
Raški	0.02	2.35	0.16	2.75	N/A	N/A	N/A	N/A
Šumadijski	0.53	0.63	0.04	6.66	N/A	N/A	N/A	N/A
Toplički	0.08	1.78	0.10	1.20	N/A	N/A	N/A	N/A
Zaječarski	0.02	2.41	0.13	2.93	N/A	N/A	N/A	N/A
Zlatiborski	0.01	2.75	0.24	1.66	N/A	N/A	N/A	N/A

## Appendix H

Parameters of the MK test for colorectal cancer at the significance level of 0.05 by county from 1999 to 2021 in the study area (*p*-value of the significance test, Z-standardised test statistics, s-so-called Sen’s slope, b-intercept).

**County**	**Colorectal Cancer**							
	**Incidence**				**Mortality**			
	***p***	**Z**	**s**	**b**	***p***	**Z**	**s**	**b**
Beogradski	0.01	−2.46	−0.40	26.10	0.03	−2.14	−0.10	12.95
Borski	8.99 × 10^−6^	4.44	0.77	9.27	0.01	2.49	0.18	7.22
Braničevski	0.11	1.58	0.20	14.40	0.41	0.82	0.08	7.84
Jablanički	3.36 × 10^−3^	2.93	0.52	10.68	0.15	−1.43	−0.10	9.60
Kolubarski	0.01	2.77	0.41	19.44	0.94	−0.08	−0.01	11.18
Mačvanski	0.02	2.35	0.40	17.80	0.62	−0.50	−0.04	11.69
Moravički	0.27	1.11	0.15	18.29	0.44	−0.77	−0.07	8.03
Nišavski	0.21	1.24	0.15	18.05	0.79	0.26	0.02	8.93
Pčinjski	0.04	2.04	0.45	11.25	0.08	−1.75	−0.10	9.40
Pirotski	0.02	2.35	0.68	16.42	0.07	1.80	0.15	6.90
Podunavski	1.00	0	−0.02	20.08	0.38	−0.87	−0.08	11.62
Pomoravski	0.14	1.48	0.25	14.25	0.04	−2.09	−0.22	11.88
Rasinski	3.25 × 10^−4^	3.59	0.65	13.78	0.85	0.18	0.02	10.59
Raški	0.15	1.45	0.20	16.60	0.60	−0.53	−0.03	8.70
Šumadijski	0.22	1.22	0.18	21.77	0.96	−0.05	0	9.30
Toplički	0.79	−0.26	−0.05	17.65	0.02	−2.27	−0.15	9.20
Zaječarski	0.10	1.64	0.28	18.38	0.07	−1.82	−0.15	13.15
Zlatiborski	0.01	2.80	0.36	13.14	0.44	0.77	0.07	8.73
