# Supplementary material for: Spatial–Temporal Trends of Cancer Among Women in Central Serbia, 1999–2021: Implications for Disaster and Public Health Preparedness
Source: Healthcare (Basel). 2025 Aug 30;13(17):2169. doi: 10.3390/healthcare13172169 (PMC12427793; doi:10.3390/healthcare13172169)
Supplement: Supplementary file 1 [file healthcare-13-02169-s001.zip › healthcare-3744205-supplementary.pdf]

Table S1. STROBE Statement

	Item No.	Page No.	Relevant text from manuscript
**Title and abstract**	1	1	Spatial-Temporal Trends of Cancer Among Women in Central Serbia, 1999–2021: Implications for Disaster and Public Health Preparedness
		1	Background/Objectives: Cancer is a major public health burden in Serbia and a factor influencing long-term disaster readiness by straining health system capacity. This study examined spatial and temporal trends in incidence and mortality for eight major cancers among women in Central Serbia (1999-2021) to inform targeted prevention and preparedness strategies. Methods: Standardized rates from national datasets were analysed using the Mann-Kendall trend test and Sen's slope estimator. Geographic disparities were mapped in ArcGIS Pro 3.2. Mortality trends were assessed only for statistically reliable series. Results: Breast cancer incidence increased in six counties, while cervical cancer declined in several areas, likely reflecting screening success. Colorectal, bladder, pancreatic, and lung and bronchus cancers showed rising incidence; lung and bronchus cancer mortality increased in 16 counties, indicating growing demand for chronic respiratory care. These shifts may reduce surge capacity during disasters by increasing the baseline burden on healthcare infrastructure. Regional disparities highlight uneven system resilience. Conclusions: Aligning cancer control measures – especially for high-burden cancers like lung – with emergency preparedness frameworks is essential to strengthen health system resilience, particularly in re-source-limited regions.
**Introduction**			
Background/rationale	2	3	According to the World Cancer Research Fund, Serbia is among the countries with high cancer rates, ranking 60th globally in newly diagnosed cancer cases among women and 57th in cancer-related mortality in 2022. The literature highlights various ongoing challenges in cancer care following disasters, including interruptions in treatment protocols, a lack of qualified healthcare personnel, and constraints in health system infrastructure
Objectives	3	4	This study aims to analyse trends in eight types of cancer – breast, cervical, uterine, colorectal, bladder, ovarian, pancreatic, and lung and bronchial – in the female population (1999 to 2021) across eighteen counties in Central Serbia. The study does not investigate the causes of cancer in women but instead focuses on analysing temporal trends.
**Methods**			
Study design	4	1	Standardized rates from national datasets were analysed using the Mann-Kendall trend test and Sen's slope estimator. Geographic disparities were mapped in ArcGIS Pro 3.2. Mortality trends were assessed only for statistically reliable series.
Setting	5	4	National datasets of the cancer rates in the female population (1999 to 2021) across eighteen counties in Central Serbia.
Participants	6	1	Standardized rates from national datasets of the cancer rates in the female population – incidence and mortality.

Variables	7	4	Outcomes: cancer incidence and mortality rates. Predictors/confounders not directly modelled; study focused on spatial-temporal patterns.
Data sources/ measurement	8	4	Cancer data were obtained from publicly available reports published by the Institute of Public Health of Serbia, "Dr Milan Jovanović Batut," specifically "Cancer Incidence and Mortality in Central Serbia from 1999–2015" and "Malignant Cancers in the Republic of Serbia 2016–2021.". Geographical boundaries from official administrative datasets. Methods: GIS (ArcGIS Pro), statistical (pymannkendall).
Bias	9	4	Registry data covers whole population, reducing selection bias. Potential underreporting acknowledged as limitation.
Study size	10	1, 4	Study included all available cancer cases from registry (1999–2021) for aforementioned cancers; no sampling performed.
Quantitative variables	11	4	Incidence and mortality counts aggregated by year and county. Standardised rates calculated. Grouped by cancer type and period.
Statistical methods	12	5-6	Mann-Kendall test for temporal trends. No imputation for missing data (registry-based). Subgroup analyses: by cancer type.
**Results**			
Participants	13	4	All registered cases in women in 1999–2021 included.
Descriptive data	14	7	Descriptive data include general population statistics regarding population number and average age by sex. Data used in the geospatial database were downloaded from open data of the National Spatial Data Infrastructure.
Outcome data	15	8-22, 27-31	Outcome data presented as incidence and mortality trends with statistical test results and maps.
Main results	16	27-31	Trends reported with p, z, s, and b statistics at the significance level of 0.05 – 95% CI (p – p-value of the significance test, z – standardised test statistics, s – so-called Sen's slope, b – intercept).
Other analyses	17		Analyses stratified by cancer type. No sensitivity analysis conducted due to complete registry data.
**Discussion**			
Key results	18	20, 22	MK analysis revealed an increasing incidence of breast cancer in six counties. In contrast, a decreasing trend in cervical cancer incidence and mortality rates was observed in several regions, reflecting possible progress in prevention and early detection. However, increasing trends for colorectal, bladder, pancreatic cancer, and lung and bronchus cancer incidence in multiple counties suggest areas requiring targeted interventions Pancreatic cancer showed increasing mortality rates in seven counties, which also represents a significant public health concern. Decreasing mortality rates for breast, cervical, and colorectal cancers in certain counties highlight potential advances in treatment and healthcare access
Limitations	19	23, 24, 25	One of the key limitations of this study is the unavailability of consistent and reliable mortality data for certain types of cancer, which restricts the application of comprehensive spatial and temporal analyses. Despite these scientific imperatives, limited financial resources and infrastructural capacities persist as significant barriers to the implementation of advanced cancer research in Serbia. Despite their advantages, the Mann–Kendall test and Sen's slope

			estimator have notable limitations. Both methods assume monotonic trends and may fail to detect non-linear, cyclical, or abrupt changes in cancer incidence, which can arise from sudden policy changes, environmental exposures, or advances in screening technologies.
Interpretation	20	21, 22, 24, 26	Mihalj et al. (2024) linked mining activities to an increase in bronchial carcinoma incidence from 2010 to 2020, identifying Borski County as the most affected area. Similarly, our MK analysis confirmed an increasing trend in lung and bronchial cancer cases among women in this county.<br>According to Sipetić-Grujičić et al. (2013), breast cancer was among the most prevalent malignancies affecting women in Central Serbia during the period 1999–2009. Furthermore, the study conducted by Stojanović et al. (2022), which analysed the most common gynaecological cancers between 2003 and 2018, revealed a significant upward trend in the incidence of these malignancies across Central Serbia from 2012 to 2018. Specifically, a marked increase in uterine cancer incidence was observed between 2014 and 2018, along with a rise in ovarian cancer cases from 2012 to 2018. In contrast, cervical cancer showed a slight downward trend in incidence from 2003 to 2015, followed by a marginal increase thereafter. When compared with the findings of our study, we observed an increasing trend in the incidence of uterine cancer in two counties and ovarian cancer in one county. Marković-Denić et al. (2006) reported increasing mortality trends for both sexes in Central Serbia between 1985 and 2002, particularly for lung and colorectal cancers. Kričković et al. (2025) conducted a spatiotemporal and trend analysis of cancer incidence among men in Central Serbia. In (2025) found rising incidence rates among men in several cancer types, with the broadest spatial increases for colorectal and pancreatic cancers. In contrast, our analysis of the female population revealed a wider distribution of increased incidence for lung and bronchus, bladder, and pancreatic cancers. Mortality trends also differed by sex, with colorectal cancer mortality more prevalent in men and pancreatic cancer mortality more widespread among women. For lung and bronchus cancer mortality, both studies identified the same counties with persistent increases or decreases, indicating stable geographic patterns over time. These results highlight the need for sex-specific cancer surveillance and sustained, regionally tailored public health interventions to address persistent high-mortality areas.<br>Incorporating cancer surveillance and spatial health analysis into national risk assessments and early warning systems is essential. Identifying clusters of chronic disease burden can aid in prioritising resources, enhancing community-level preparedness planning, and strengthening overall public health resilience.<br>Linking these findings with emergency preparedness is critical for effective policy action. Increasing trend cancer regions should be prioritised in disaster response planning, with measures such as maintaining uninterrupted oncology services, securing treatment supply chains, and deploying mobile care units during crises. Integrating cancer surveillance into national preparedness frameworks would allow timely, evidence-based resource allocation, reduce treatment disruptions, and strengthen overall health-system resilience in the face of natural hazards, pandemics, or other emergencies.

			This study highlights the importance of integrating cancer surveillance into broader disaster risk reduction and public health resilience strategies. As chronic diseases increasingly influence the health vulnerabilities faced by populations, particularly women, their inclusion in national preparedness and adaptation plans becomes essential. The increasing incidence of cancer, especially lung, pancreatic, and colorectal cancers, highlights the necessity for improved screening, environmental monitoring, and access to early diagnostics in areas with high risk.
Generalisability	21	26	From a scientific perspective, this study demonstrates the value of applying non-parametric statistical methods and geospatial technologies to long-term epidemiological data, providing a replicable framework for analysing cancer trends in other regions with similar data limitations. It contributes to the expanding field of spatial epidemiology by bridging gaps between medical research, environmental science, and public health planning.
**Other information**			
Funding	22	26	This study was supported by the Program of Cooperation with the Serbian Scientific Diaspora – Joint Research Projects – DIASPORA 2023, from the Science Fund of the Republic of Serbia, under the project LAMINATION (The Loess Plateau Margins: Towards Innovative Sustainable Conservation), Project number: 17807 and the support of the Ministry of Science, Technological Development and Innovation of the Republic of Serbia (Grants No 451-03-137/2025-03/ 200125 & 451-03-136/2025-03/200125 and 451-03-136/2025-03/200091). Furthermore, this study was supported by the Scientific-Professional Society for Disaster Risk Management and ProSafeNet – The Global Hub for Safety, Security, Risk & Emergency Professionals and Scientists (https://prosafenet.com/).
